# Remote Activation of Spinal TRPV1 by Magnetic Nanocubes Confers Cardioprotection Against Myocardial Ischemia‐Reperfusion Injury

**DOI:** 10.1002/advs.202520852

**Published:** 2025-12-12

**Authors:** Xueying Cheng, Shuangyu Liu, Yu Zhang, Kang Peng, Muge Qile, Chao Wu, Mengyun Dou, Liu Liu, Na Yang, Rui Liu, Guiyang Zhang, Liangping Ni, Gaolin Liang, Fang Yang, Ye Zhang, Shufang He

**Affiliations:** ^1^ Department of Anesthesiology and Perioperative Medicine The Second Affiliated Hospital of Anhui Medical University Key Laboratory of Anesthesiology and Perioperative Medicine of Anhui Higher Education Institutes Anhui Medical University Hefei 230601 China; ^2^ School of Biological Sciences and Medical Engineering State Key Laboratory of Digital Medical Engineering Southeast University Nanjing 201196 China; ^3^ School of Basic Medical Sciences Anhui Medical University Hefei 230032 China; ^4^ Department of Radiology The Second Affiliated Hospital of Anhui Medical University Medical Imaging Research Center Anhui Medical University Hefei 230601 China; ^5^ State Key Laboratory of Bioelectronics School of Biological Sciences and Medical Engineering Southeast University Nanjing 210096 China

**Keywords:** iron oxide nanocubes, magnetothermal effect, myocardial ischemia‐reperfusion injury, spinal cord, transient receptor potential vanilloid 1

## Abstract

The heat‐sensitive transient receptor potential vanilloid 1 (TRPV1), which is highly expressed on cardiac sensory neurons, reportedly plays a crucial role in transmitting nociceptive signals from the heart to the spinal cord during myocardial ischemia and reperfusion. Here, iron oxide nanocubes (FeNCs) are developed that are conjugated with an antibody against the extracellular portion of TRPV1, and they are named FeNCs‐TRPV1. In F11 cell line and primary dorsal root ganglion neurons, FeNCs‐TRPV1 specifically activate TRPV1 channels and trigger Ca^2+^ influx through magnetothermal effect under an alternating current magnetic field (ACMF). Intraspinally injected FeNCs‐TRPV1 induced TRPV1 desensitization in rats exposed to repetitive and transient ACMF before ischemia, resulting in the inhibition of TRPV1‐mediated Ca^2+^ signaling and neuropeptide release in the spinal cord during myocardial ischemia and reperfusion. Consequently, FeNCs‐TRPV1 reduce cardiac injury and ventricular arrhythmia, enhance the activity of prosurvival kinases, and inhibit myocardial cell apoptosis. These findings suggest that magnetic nanomaterials‐mediated remote regulation of spinal TRPV1 can be a novel non‐invasive neuromodulation therapy for the treatment of myocardial ischemia‐reperfusion (IR) injury.

## Graphical abstract

Fe‐based magnetic nanocubes conjugated with TRPV1 antibodies (FeNCs‐TRPV1) are developed for the specific targeting of TRPV1 channels. Intraspinally injected FeNCs‐TRPV1 induces TRPV1 desensitization in rats exposed to repetitive and transient ACMF. This results in a significant reduction of myocardial ischemic injury, and the cardioprotection is associated with reduced neuropeptides release, decreased cardiac sympathetic efferent activity, and enhanced cardiac cell survival.

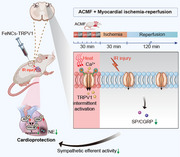

## 1 Introduction

Ischemic heart disease is a leading cause of disability and mortality worldwide.^[^ ^1^, ^2^ ^]^ Following an attack of myocardial infarction, timely reperfusion has emerged as the most effective treatment strategy, substantially reducing mortality associated with ischemic heart disease.^[^ ^3^, ^4^ ^]^ However, the occurrence of myocardial ischemia‐reperfusion (IR) injury may offset the beneficial effects of reperfusion therapy,^[^ ^5^ ^]^ and exacerbate ischemic damage, thereby increasing the risk of arrhythmia and death.^[^ ^6^, ^7^ ^]^ Effective clinical interventions specifically targeting myocardial IR injury are lacking. It is important to develop potential strategies for the prevention of myocardial damage.

Endogenous mediators generated during the phases of ischemia and reperfusion, such as adenosine, protons, bradykinin, and lipids, can stimulate cardiac afferent neurons that express transient receptor potential vanilloid 1 (TRPV1).^[^ ^8^, ^9^ ^]^ The hyperactivation of TRPV1 on cardiac sensory neurons during ischemia and reperfusion procedures can lead to the release of substance P (SP) and calcitonin gene‐related peptide (CGRP) from nerve endings to the spinal cord, which in turn enhances the spinal cardiogenic sympathetic reflex and aggravates myocardial injury.^[^ ^10^, ^11^, ^12^ ^]^ Our recent studies demonstrate that the inhibition of TRPV1 activation in the dorsal root ganglion (DRG) or spinal cord mitigates myocardial IR injury and the mechanisms involving the reduced release of SP and CGRP in the spinal cord and subsequently the decreased cardiac sympathetic activity.^[^ ^13^, ^14^ ^]^ This suggests that the spinal TRPV1 channel can serve as an important therapeutic target for preventing myocardial ischemic injury.

On the other hand, studies have shown that TRPV1 activation is crucial for ischemic preconditioning (IPC)‐mediated myocardial protection. IPC refers to stimulating the myocardium with several brief episodes of ischemia and reperfusion before prolonged ischemia, which can increase the myocardium's tolerance to subsequent longer periods of ischemia and thereby reduce myocardial IR injury.^[^ ^15^ ^]^ It is reported that knockout of the TRPV1 gene impairs IPC‐mediated cardioprotection owing to the reduced release of SP and/or CGRP from cardiac sensory nerve endings.^[^ ^16^, ^17^ ^]^ Additionally, the cardioprotection induced by remote IPC (via pressure cuff on limb) is also dependent on TRPV1 activation.^[^ ^18^, ^19^ ^]^ The controversial effects of TRPV1 in myocardial IR injury could be due to the different sensitivity of the channel. It is noted that repeated stimulation of its agonist can lead to the desensitization of the TRPV1 channel.^[^ ^20^ ^]^ Unfortunately, although the myocardial protective effect of IPC has been proven in a large number of animal experiments, its clinical application is greatly limited owing to the fact that IPC is an invasive operation and is not ethically feasible. Therefore, a non‐invasive approach targeting TRPV1 may present a promising strategy for cardioprotection.

TRPV1 is a heat‐sensitive nonspecific cation channel that opens when the temperature exceeds 43 °C.^[^ ^21^ ^]^ The opening of TRPV1 channels allows the influx of cations, especially Ca^2+^, which in turn activates downstream signaling pathways.^[^ ^22^ ^]^ Leveraging the thermosensitive characteristic of TRPV1, gold nanorod‐, Cu‐ or Fe‐based nanomaterials that bind to TRPV1 have been reported to raise the local temperature and induce the opening of the TRPV1 channel upon exposure to near‐infrared light,^[^ ^23^, ^24^, ^25^, ^26^, ^27^, ^28^ ^]^ radio wave,^[^ ^29^ ^]^ or alternating current magnetic field (ACMF).^[^ ^30^, ^31^ ^]^ These approaches have been shown to remotely regulate gene expression, trigger signal transduction, or deliver drugs, achieving the purpose of reducing atherosclerosis and thrombosis,^[^ ^26^, ^28^ ^]^ inhibiting cell apoptosis and inflammation,^[^ ^25^, ^30^ ^]^ or treating neurodegenerative diseases.^[^ ^24^, ^31^ ^]^ However, it is unknown whether nanomaterial‐mediated remote regulation of TRPV1 channels can lessen the severity of myocardial ischemic injury.

Among various nanomaterials, iron oxide‐based nanoparticles exhibit good biosafety, biodegradability, and clinical practicability. Notably, several iron oxide nanoparticles have been approved by the Food and Drug Administration (USA) for the treatment of iron‐deficiency anemia, chronic kidney disease, and to enhance magnetic resonance imaging.^[^ ^32^, ^33^, ^34^ ^]^ In this study, we synthesized iron oxide nanocubes (FeNCs) that were conjugated to an antibody against the extracellular portion of TRPV1, named as FeNCs‐TRPV1, to specifically bind to the extracellular region of the TRPV1 channel. By utilizing the magnetothermal effect, we demonstrated that FeNCs‐TRPV1 induced the activation of the TRPV1 channel and Ca^2+^ influx in F11 cells and primary DRG neurons. In vivo, intraspinally injected FeNCs‐TRPV1 mediated remote activation of spinal TPRV1 channels through repetitive ACMF exposure in a preconditioning manner, which led to desensitization of TRPV1 channels and a significant reduction in cardiac injury and ventricular arrhythmia during myocardial IR.

## 2 Results

### 2.1 Preparation and Characterization of FeNCs‐TRPV1

**Figure 1:**
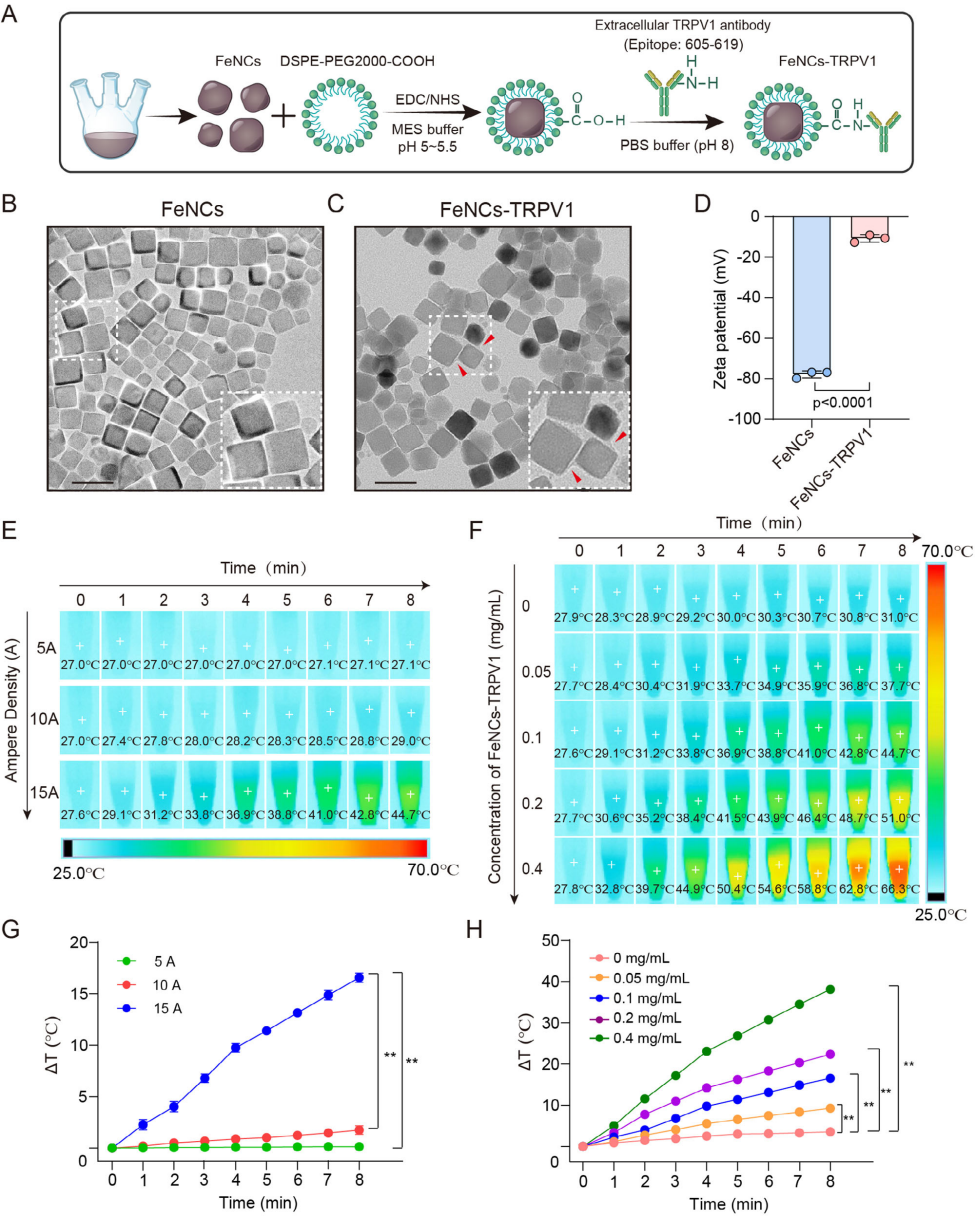
Preparation and characterization of FeNCs‐TRPV1. A) Schematic diagram of the synthesis of FeNCs‐TRPV1. B,C) Transmission electron microscopy images of FeNCs and FeNCs‐TRPV1. Scale bar = 50 nm. D) Zeta potential results of FeNCs and FeNCs‐TRPV1. Data represent the mean ± standard deviation (SD) from three independent experiments. Statistical analysis was performed using an unpaired *t*‐test. E) Infrared thermal images of FeNCs‐TRPV1 (0.1 mg mL^−1^) upon exposure to ACMF at different magnetic densities. F) Infrared thermal images of FeNCs‐TRPV1 at different concentrations upon exposure to ACMF at 15 A. G) Optical fiber thermometer recording of FeNCs‐TRPV1 (0.1 mg mL^−1^) upon exposure to ACMF at different magnetic densities. H) Optical fiber thermometer recording of FeNCs‐TRPV1 at different concentrations upon exposure to ACMF at 15 A. Δ*T*: Temperature rise. Data represent the mean ± SD from four independent experiments in G and H, and statistical analysis was performed using repeat measurement two‐way analysis of variance (ANOVA) followed by Tukey's test. ^**^ *p* <0.01.

FeNCs were initially prepared using a traditional high‐temperature pyrolysis method, and then the PEGylated long‐circulating lipid DSPE‐PEG2000 molecules were coupled to the FeNCs surface. Subsequently, a TRPV1 extracellular antibody was conjugated to the surface of FeNCs via a condensation reaction between the carboxyl group of FeNCs and the N‐terminal amino group of the antibody, resulting in the formation of the FeNCs‐TRPV1 (**Figure** **1**A). Transmission Electron Microscope (TEM) images revealed that both FeNCs and FeNCs‐TRPV1 were evenly dispersed, and there was no noticeable difference in their morphological characteristics. The enlarged image showed a membrane‐like coating on the surface of FeNCs‐TRPV1, which indicates the successful conjugation of the TRPV1 antibody (Figure 1C). In contrast, FeNCs displayed well‐defined edges (Figure 1B). Moreover, the zeta potential of FeNCs changed from −77.81 ± 1.70 mV to a less negative value after conjugation with TRPV1 antibody (FeNCs‐TRPV1: −10.76 ± 1.81 mV), further confirming successful conjugation (Figure 1D).^[^ ^35^ ^]^ When analyzed by SDS‐PAGE electrophoresis, FeNCs‐TRPV1, due to their large size, was retained in the loading well, and the bound antibody produced a blue‐stained band distinct from residual dye staining. In contrast, FeNCs, without antibody showed no such band. Conversely, the TRPV1 antibody (TRPV1 Ab) alone exhibited a blue‐stained band at 150 kDa (Figure S1, Supporting Information).

The size distribution and concentration of FeNCs and FeNCs‐TRPV1 were assessed using Nanoparticle Tracking Analysis (NTA). As shown in Figure S2A (Supporting Information), the FeNCs sample had a concentration of 1.9 × 10^12^ particles mL^−1^ with a mean diameter of 77.5 ± 35.6 nm. In contrast, the FeNCs‐TRPV1 exhibited a concentration of 2.0 × 10^12^ particles mL^−1^ with a diameter range of 100.4 ± 39.5 nm (Figure S2B, Supporting Information). Furthermore, the bicinchoninic acid (BCA) protein assay was employed to quantify the antibody concentration in the FeNCs‐TRPV1 solution.^[^ ^36^ ^]^ Based on the standard curve, the antibody concentration was determined to be 128 ± 13 µg mL^−1^, which corresponds to ≈ 64.0 × 10^−12^ µg of antibody bound per individual nanocube.

To evaluate the efficiency of magnetothermal conversion, a FeNCs‐TRPV1 solution (0.1 mg mL^−1^, based on the total particle mass) was exposed to an ACMF at current intensities of 5, 10, or 15 A for up to 8 min. Temperature changes were monitored using an infrared thermal imaging camera and an optical fiber thermometer. A time‐dependent magnetothermal effect was observed only at 15 A (Figure 1E). Under this condition, the 0.1 mg mL^−1^ FeNCs‐TRPV1 solution reached a temperature increase (Δ*T*) of 11.4 ± 0.32 °C after 5 min of exposure (Figure 1G). Moreover, when tested at different concentrations under the 15 A ACMF, the FeNCs‐TRPV1 solution exhibited a concentration‐dependent enhancement in heating (Figure 1F,H). The magnetothermal performance remained consistent across multiple 5‐min ACMF exposure cycles (Figure S3, Supporting Information). These results confirm that FeNCs‐TRPV1 can effectively generate heat under ACMF stimulation, and the heating effect can be controlled by adjusting either the ACMF current intensity or the nanoparticle concentration.

### 2.2 FeNCs‐TRPV1 Specifically Bound to TRPV1 Channel in rTRPV1‐Transfected F11 Cells

**Figure 2:**
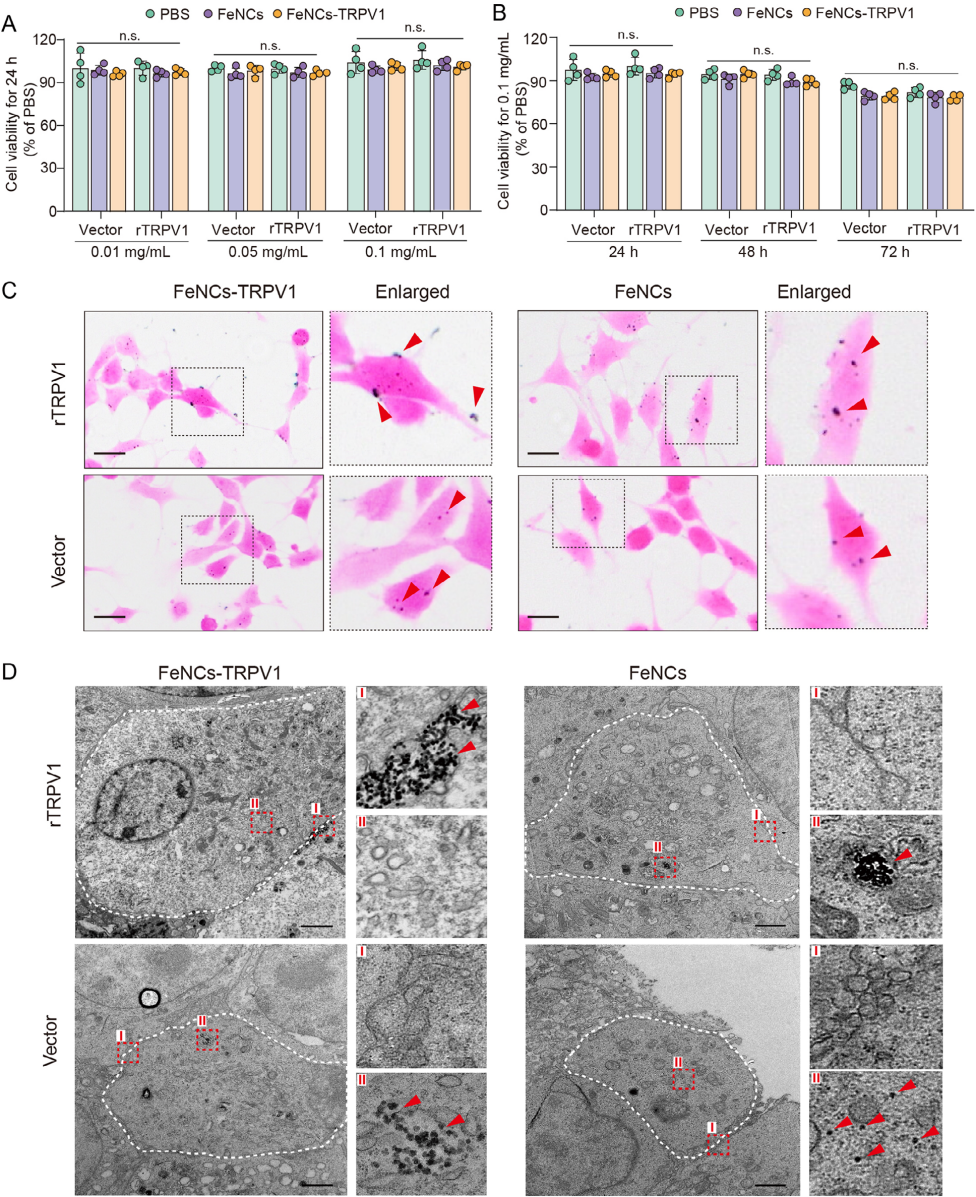
Specific binding of FeNCs‐TRPV1 to TRPV1‐expressed F11 cells. A) Cell viability of F11 cells incubated with FeNCs or FeNCs‐TRPV1 at different iron concentrations for 24 h. B) Cell viability of F11 cells incubated with FeNCs or FeNCs‐TRPV1 at the iron concentration of 0.1 mg mL^−1^ for 24, 48, and 72 h. PBS served as a control. Data represent the mean ± SD of four independent experiments and were analyzed with the two‐way ANOVA followed by Tukey's test. C) Prussian blue staining images of the transfected F11 cells incubated with FeNCs or FeNCs‐TRPV1 for 2 h. Red arrows point to blue‐stained iron nanocubes. Scale bar = 20 µm. D) Representative transmission electron microscopy images of F11 cells incubated with 0.1 mg mL^−1^ FeNCs‐TRPV1 or FeNCs for 2 h. The white dotted outline represents the cell membrane. Right panels are enlarged images showing cell surface area (I) and intracellular area (II). Red arrows indicate the localization of nanocubes. Scale bar = 2 µm.

To examine whether FeNCs‐TRPV1 can specifically bind to the TRPV1 channel, we utilized F11 cells that are transfected with the rTRPV1 plasmid or the vector. The successful transfection was verified by the expression of Myc‐tag (implying TRPV1 expression) in the rTRPV1‐transfected F11 cells but not in the vector‐transfected cells (Figure S4A–C, Supporting Information). To evaluate the impact of the nanocubes on cell viability, the transfected F11 cells were incubated with FeNCs or FeNCs‐TRPV1 (each 0.01, 0.05, and 0.1 mg mL^−1^, respectively) for 24 h. Moreover, the transfected F11 cells were incubated with FeNCs or FeNCs‐TRPV1 (each 0.1 mg mL^−1^) for 24, 48, and 72 h, respectively. No significant difference in cell viability was observed among groups, which suggests that the nanocubes exhibit no cytotoxicity to cells (**Figure** **2**A,B).

After incubation with FeNCs‐TRPV1 or FeNCs, the transfected cells were collected to examine the localization of the nanocubes by using Prussian blue staining and TEM. In the rTRPV1‐transfected cells, FeNCs‐TRPV1 were mainly distributed on the cell surface, while FeNCs were localized in the cytoplasm. In contrast, both FeNCs‐TRPV1 and FeNCs were localized in the cytoplasm of vector‐transfected cells (Figure 2C). Furthermore, TEM images revealed that FeNCs‐TRPV1 were gathered on the cell membrane of rTRPV1‐transfected cells, whereas FeNCs were internalized into the cytoplasm of these cells (Figure 2D). By comparison, both FeNCs and FeNCs‐TRPV1 were internalized into the cytoplasm of the vector‐transfected cells (Figure 2D). These results indicate that FeNCs‐TRPV1 can specifically bind to TRPV1 channels on the cell surface, which might facilitate the activation of TRPV1 channels upon ACMF exposure.

### 2.3 FeNCs‐TRPV1 Promotes TRPV1‐Mediated Ca^2+^ Influx Through Magnetothermal Effect

**Figure 3:**
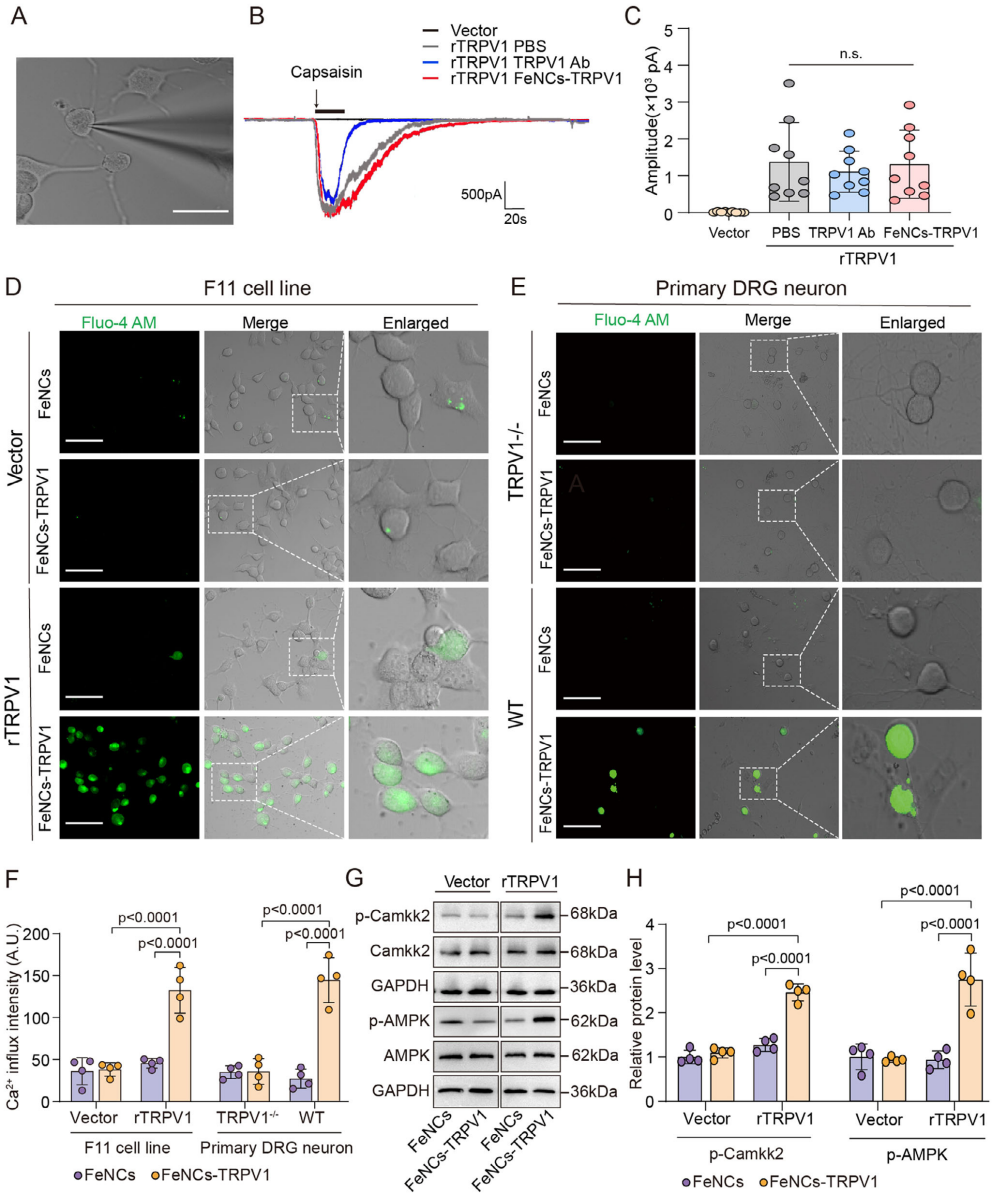
FeNCs‐TRPV1 promotes TRPV1‐mediated Ca^2+^ influx through magnetothermal effect. A) Representative image of F11 cells under patch clamp recording. Scale bar = 25 µm. B) Representative traces of capsaicin‐induced inward currents in TRPV1‐transfected F11 cells incubation with PBS (gray), TRPV1 antibody (TRPV1 Ab, blue), or FeNCs‐TRPV1 (red). The vector‐transfected F11 cells served as the blank control (black). C) Quantitative analysis of the amplitude of capsaicin‐induced inward currents. Data represent the mean ± SD. Statistical analysis was performed using one‐way ANOVA followed by Tukey's test (*n* = 9 cells per group from three independent experiments). D,E) Representative images for intracellular Ca^2+^ signal detected by Fura‐4AM fluorescent probe in F11 cells (D) or primary DRG neurons (E). Scale bar = 50 µm. F) Fluorescent intensity (arbitrary unit, A.U.) was quantified based on four independent experiments. G) Representative immunoreactive bands of phosphorylated Camkk2 (p‐Camkk2), Camkk2, phosphorylated AMPK (p‐AMPK), AMPK, and the reference gene GAPDH. H) The relative levels of p‐Camkk2 and p‐AMPK were expressed as ratios of their phosphorylated forms to total protein, following normalization to GAPDH, and the value in the FeNCs‐treated vector group was assigned as 1. Data represent the mean ± SD from four independent experiments. The analysis was performed using the two‐way analysis of ANOVA followed by Tukey's test.

To determine whether the activity of the TRPV1 channel is affected by nanocube binding, we employed the single‐cell patch clamp technique for measuring capsaicin‐induced TRPV1 currents. **Figure** **3**A shows a representative image of F11 cells under patch clamp recording. Following 1 µm capsaicin stimulation for 30 s, rTRPV1‐transfected cells exhibited a distinct inward current across all groups, while vector‐transfected cells showed no response (Figure 3B). No significant differences were observed in the amplitude of capsaicin‐induced current among rTRPV1‐transfected cells incubated with PBS, TRPV1 Ab, or FeNCs‐TRPV1 (Figure 3C).

The activation of TRPV1 on the cell membrane leads to the channel opening and Ca^2+^ influx.^[^ ^37^ ^]^ To investigate whether FeNCs‐TRPV1 can induce Ca^2+^ influx by activating TRPV1 upon ACMF exposure, two experimental cell models (F11 cells and primary DRG neurons) were used in this study. The intracellular ionized Ca^2+^ level was determined by using a cell‐permeant fluorescent dye Fluo‐4 AM.^[^ ^38^ ^]^ Based on the results of magnetothermal conversion, the magnetic intensity was set at 15 A, and the exposure duration was maintained for 5 min. After exposure to ACMF, FeNCs‐TRPV1 elicited Ca^2+^ influx in only the rTRPV1‐transfected cells, as evidenced by the strong green fluorescence (Figure 3D). In contrast, FeNCs did not increase intracellular Ca^2+^ signal in either the rTRPV1‐transfected or the vector‐transfected cells (Figure 3D). Quantification of fluorescence intensity revealed that FeNCs‐TRPV1 led to a 2.9‐fold rise in intracellular Ca^2+^ level compared to FeNCs in rTRPV1‐transfected cells exposed to ACMF (Figure 3F, *p* <0.0001). Additionally, in the absence of the ACMF exposure, FeNCs‐TRPV1 alone has no effects on intracellular Ca^2+^ signal in either rTRPV1‐ or vector‐transfected cells (Figure S5, Supporting Information).

Moreover, primary DRG neurons were isolated from adult wild‐type (WT) and TRPV1 knockout (TRPV1^−/−^, Shanghai Model Organisms Center Inc.) mice, and the protein expression of TRPV1 was verified (Figure S4D, Supporting Information). When WT DRG neurons were treated with FeNCs‐TRPV1 and exposed to ACMF, the intracellular Ca^2+^ levels were increased by 5.45‐fold relative to those treated with FeNCs (Figure 3E,F, *p* <0.0001). In contrast, DRG neurons derived from TRPV1^−/−^ mice failed to induce Ca^2+^ influx when treated with either FeNCs‐TRPV1 or FeNCs under ACMF exposure.

To investigate whether the increase of intracellular Ca^2+^ level induced by FeNCs‐TRPV1 could promote the downstream signaling processes, we examined the activation status of calcium/calmodulin‐dependent protein kinase kinase 2 (Camkk2) and the AMP‐activated protein kinase (AMPK), which are the key downstream effectors of TRPV1‐mediated Ca^2+^ signaling.^[^ ^24^, ^26^ ^]^ Notably, when rTRPV1‐expressing F11 cells were incubated with FeNCs‐TRPV1 and then exposed to ACMF, the significant 1.93‐fold increase in p‐Camkk2 and 2.94‐fold increase in p‐AMPK protein levels were detected in comparison to cells treated with FeNCs (Figure 3G,H, *p* <0.0001). Together, these findings provide evidence that FeNCs‐TRPV1 has the capacity to induce the opening of TRPV1 channels, elicit Ca^2+^ influx, and activate downstream signaling upon exposure to ACMF.

### 2.4 FeNCs‐TRPV1 Mitigates Myocardial Injury in Rats Subjected to Repeated ACMF Exposure

**Figure 4:**
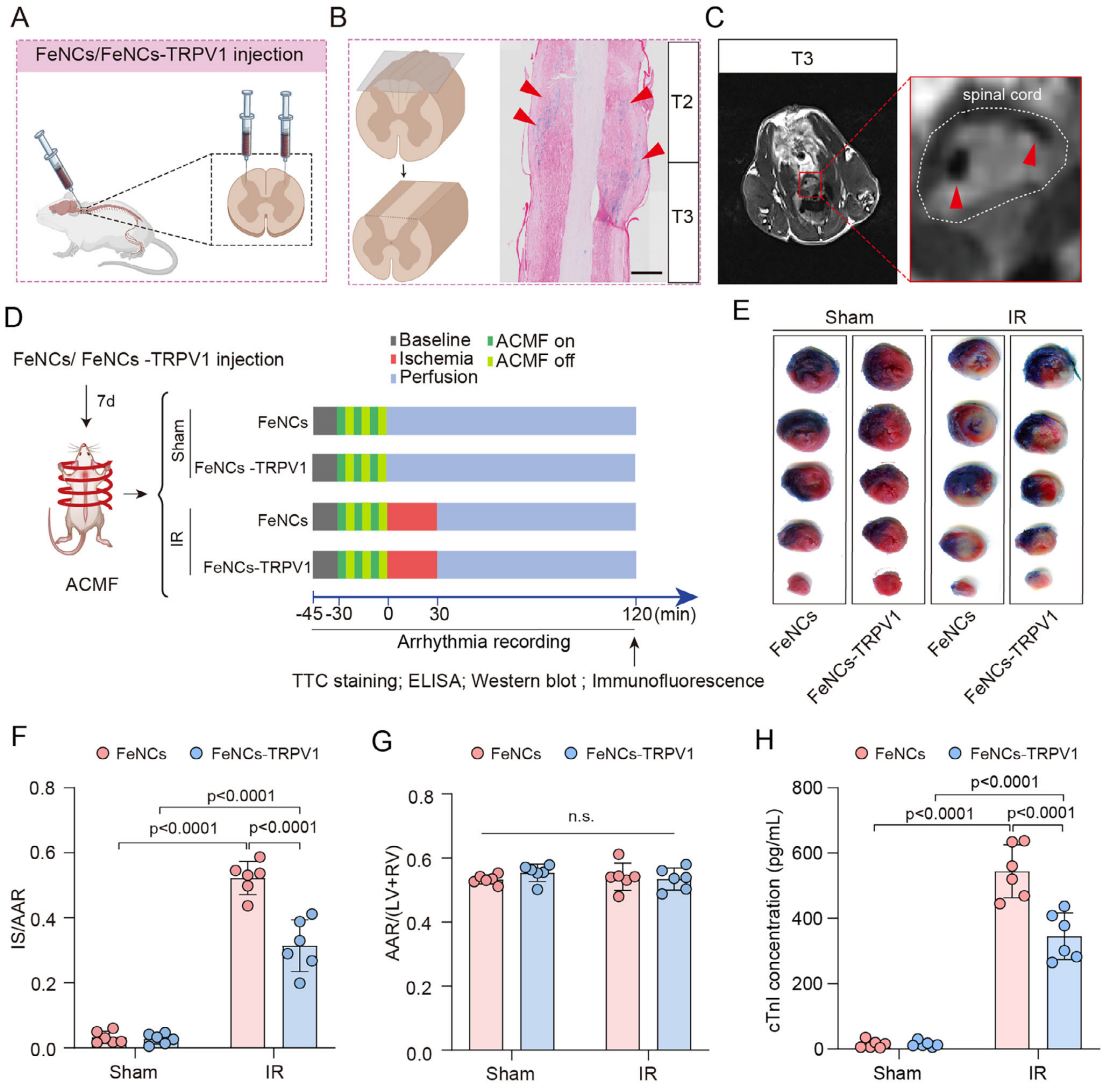
FeNCs‐TRPV1 mitigates myocardial injury in rats subjected to repeated ACMF exposure. A) Schematic diagram for intra‐spinal injection of nanocubes. B) Prussian blue staining of the longitudinally transected spinal cord. The red arrows point to the blue‐stained nanocubes. Scale bar = 500 µm. C) MRI images of the spinal cord. The white dotted outline represents the area of the spinal cord. The red arrow points to the localization of the injected nanocubes. D) Schematic diagram of the experimental procedure. E) Representative images of heart sections stained by TTC (2,3,5‐triethylenetetrazolium chloride) and Evans blue. F) Infarct size (IS) is expressed as the ratio to the area at risk (AAR). G) The ratio of AAR to left ventricular (LV) and right ventricular (RV). H) Serum cardiac troponin I (cTnI) levels measured by ELISA. Data were expressed as the mean ± SD and were analyzed using two‐way ANOVA followed by Tukey's test (*n* = 6 per group).

FeNCs or FeNCs‐TRPV1 were bilaterally injected into the T2‐T4 segments of the thoracic spinal cord as illustrated in **Figure** **4**A. The localization of nanocubes in the spinal cord was confirmed by using Prussian blue staining that can detect iron in histological sections. The spinal cord was longitudinally transected along the direction of the dorsal horn. The regions stained red on both sides are identified as the dorsal horns, with the intermediate area being the white matter zone. It was observed that the nanocubes stained blue were predominantly located within the dorsal horn region of the T2‐T3 spinal cord segments (Figure 4B). Additionally, the nanocubes were found to persist in the spinal cord on day 28 after injection (Figure S6, Supporting Information). Given that iron nanocubes possess superparamagnetic properties and can be visualized in Magnetic Resonance Imaging (MRI), we further examined the localization of the nanocubes by using a 3.0 T MRI. It was observed in living rodents that the iron nanocubes were presented in the dorsal horn region of the spinal cord, as indicated by the red arrows (Figure 4C).

After confirming the nanocubes' localization in the spinal cord, we exposed the rats injected with FeNCs or FeNCs‐TRPV1 to ACMF in a preconditioning manner (three cycles of ACMF 5 min on/5 min off) before lethal myocardial ischemia (Figure 4D), to simulate the classical IPC procedure. Representative heart sections of each group are presented in Figure 4E. In these sections, the non‐ischemic area was stained blue, the area at risk (AAR) appeared brick red, and the infarct size (IS) was white. When the FeNCs‐TRPV1 injected rats were subjected to repeated ACMF exposure prior to myocardial ischemia, they demonstrated a significantly lower IS/AAR ratio compared to those injected with FeNCs (0.31 ± 0.08 vs 0.52 ± 0.05, respectively; *p* <0.0001, Figure 4F). There was no significant difference in the ratio of AAR/ left ventricle + right ventricle (LV + RV) among each group (Figure 4G). Additionally, the serum concentration of cardiac troponin (cTnI), which serves as an indicator of the extent of cardiac injury, was found to be lower in rats injected with FeNCs‐TRPV1 compared to those injected with FeNCs (Figure 4H).

Furthermore, we confirmed the cardioprotective effects of the classical IPC, as evidenced by the reduced ratio of IS/AAR and the decreased concentration of cTnI when compared to the IR group (Figure S7A–E, Supporting Information). However, ACMF exposure had no noticeable effects on either the myocardial infarct size or the concentration of cTnI in rats without nanocubes injection (Figure S7A–E, Supporting Information). Moreover, in the postmortem analysis of rats’ liver, spleen, lung, and kidney, there were no obvious structural or cellular changes in the group of FeNCs or FeNCs‐TRPV1 compared with the Sham group by hematoxylin and eosin (H&E), which indicated that FeNCs‐TRPV1 with ACMF stimulation was safe and well tolerated in rats (Figure S8, Supporting Information).

### 2.5 FeNCs‐TRPV1 Alleviates Ventricular Arrhythmia and Promotes Cardiac Cells Survival

**Figure 5:**
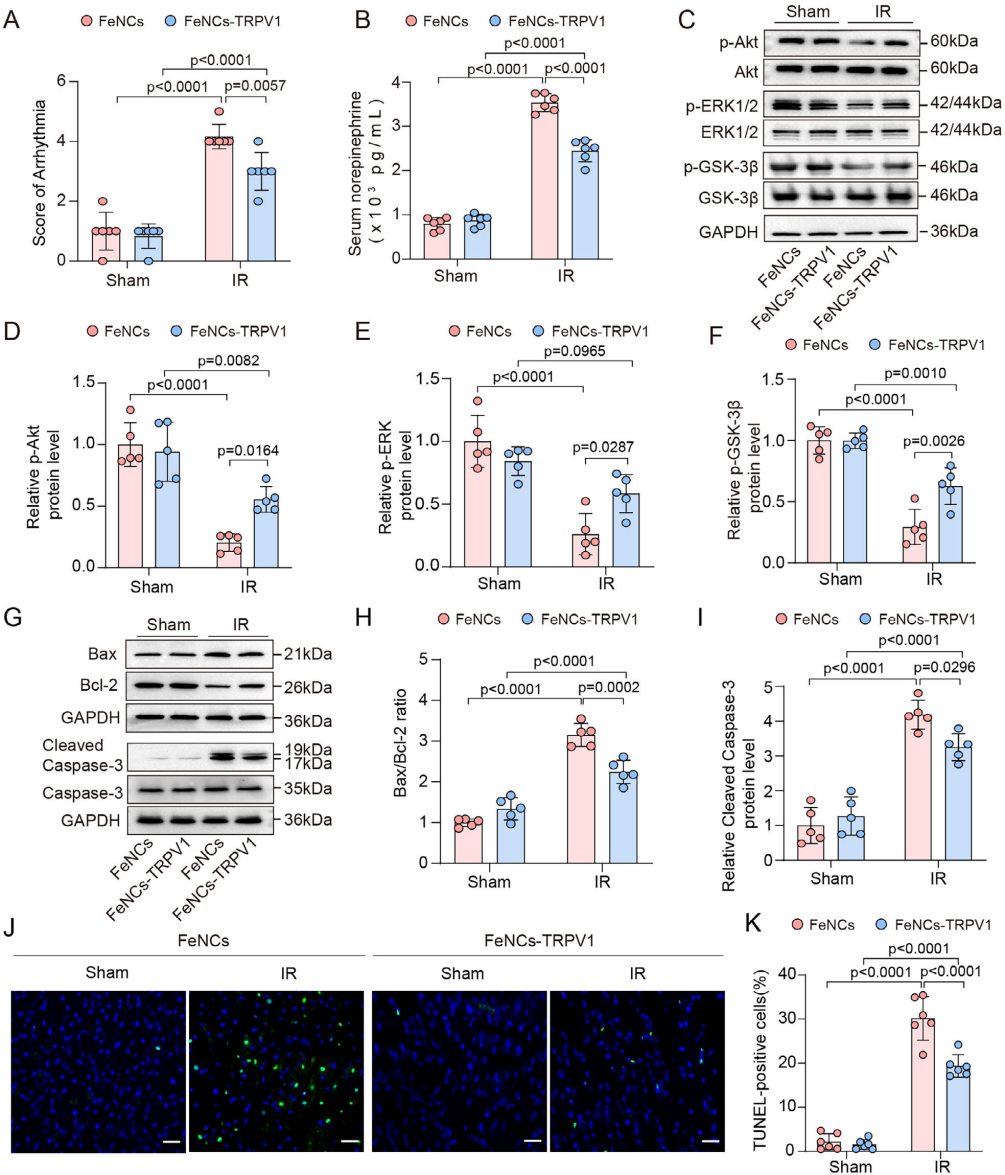
FeNCs‐TRPV1 alleviates ventricular arrhythmia and promotes cardiac cells survival. A) Arrhythmia score for each group. B) Serum Norepinephrine levels measured by ELISA for each group. C) Representative immunoreactive bands of p‐Akt/Akt, p‐ERK1/2/ERK1/2, p‐GSK‐3β/GSK‐3β, and the reference GAPDH in heart tissue samples. D–F) The relative levels of p‐Akt, p‐ERK1/2, and p‐GSK‐3β were expressed as ratios of their phosphorylated forms to total protein, following normalization to GAPDH, and the value in the FeNCs‐injected Sham group was assigned as 1. G) Representative immunoreactive bands of Bax/Bcl‐2, cleaved caspase‐3/caspase‐3, and the reference GAPDH in heart tissue samples. H,I) The relative ratios of Bax/Bcl‐2 and cleaved caspase‐3/caspase‐3 were calculated following normalization to GAPDH, the value in the FeNCs‐injected Sham group was assigned as 1. J) Representative images for TUNEL staining in the left ventricular tissues. Scale bar = 20 µm. K) The number of TUNEL‐positive apoptotic cells was quantified and expressed as a percentage of the total number of cells. Data represent the mean ± SD. Statistical analysis was performed using two‐way ANOVA followed by Tukey's test (*n* = 6 per group in A, B, *n *= 5 per group in C–I, *n *= 6 per group in J,K).

Myocardial IR injury frequently induces severe ventricular arrhythmia, potentially attributable to the enhanced activity of cardiac sympathetic efferent nerves.^[^ ^39^ ^]^ The arrhythmia events occurred during myocardial IR, including premature ventricular contraction (PVC), ventricular tachycardia (VT), and ventricular fibrillation (VF) (Figure S9, Supporting Information). Our findings revealed that, when exposure to ACMF before myocardial ischemia, the rats injected with FeNCs‐TRPV1 exhibited a significantly lower incidence of ventricular arrhythmia events (Supplemental Table S1) and decreased arrhythmia score (3.0 ± 0.6 vs 4.2 ± 0.4, respectively; *p* = 0.0057, **Figure** **5**A) compared to those injected with FeNCs. Additionally, serum norepinephrine concentration was reduced in the FeNCs‐TRPV1 group compared to the FeNCs group (2445 ± 248 vs 3535 ± 204 pg mL^−1^, respectively; *p* <0.0001, Figure 5B).

We further explored whether the cardioprotective effects of FeNCs‐TRPV1 are associated with the regulation of intramyocardial prosurvival kinases, specifically protein kinase B (Akt), extracellular signal‐regulated kinase (ERK), and glycogen synthase kinase‐3β (GSK‐3β).^[^ ^40^ ^]^ It was found that these kinases were activated by FeNCs‐TRPV1 upon exposure to ACMF. In comparison with those of rats injected with FeNCs, the protein levels of p‐Akt, p‐ERK, and p‐GSK‐3β were significantly elevated by 2.73, 2.25, and 2.14‐fold, respectively (Figure 5C–F). The activation of the prosurvival kinases is known to promote cardiac cells survival by inhibiting apoptotic signaling. Our data showed that FeNCs‐TRPV1 suppressed myocardial pro‐apoptotic signaling under ACMF exposure. Specifically, FeNCs‐TRPV1 significantly reduced IR‐increased Bax/Bcl‐2 ratio (*p* = 0.0002, Figure 5G,H) and cleaved caspase 3 protein levels (*p* = 0.0296, Figure 5G,I) compared to FeNCs upon ACMF exposure. Additionally, TUNEL staining of cardiac tissues revealed that the percentage of apoptotic cells in the FeNCs group was 30.14% after IR injury, whereas this percentage was reduced to 19.37% in the FeNCs‐TRPV1 group (*p* <0.0001, Figure 5J,K). Again, the classic IPC significantly decreased the arrhythmia score (Figure S10A, Supporting Information) and serum norepinephrine concentration (Figure S10B, Supporting Information), promoted cardiac cell survival by activating Akt/ERK/GSK‐3β and inhibiting apoptotic signaling (Figure S10C–F, Supporting Information). Nevertheless, exposure of ACMF in rats without nanocubes injection exerted no discernible effects on the arrhythmia score, serum norepinephrine concentration, the activation of prosurvival kinases, and cardiac cell apoptosis (Figure S10, Supporting Information).

### 2.6 FeNCs‐TRPV1 Inhibits Calcium‐Dependent Signaling and Neuropeptides Release in the Spinal Cord

**Figure 6:**
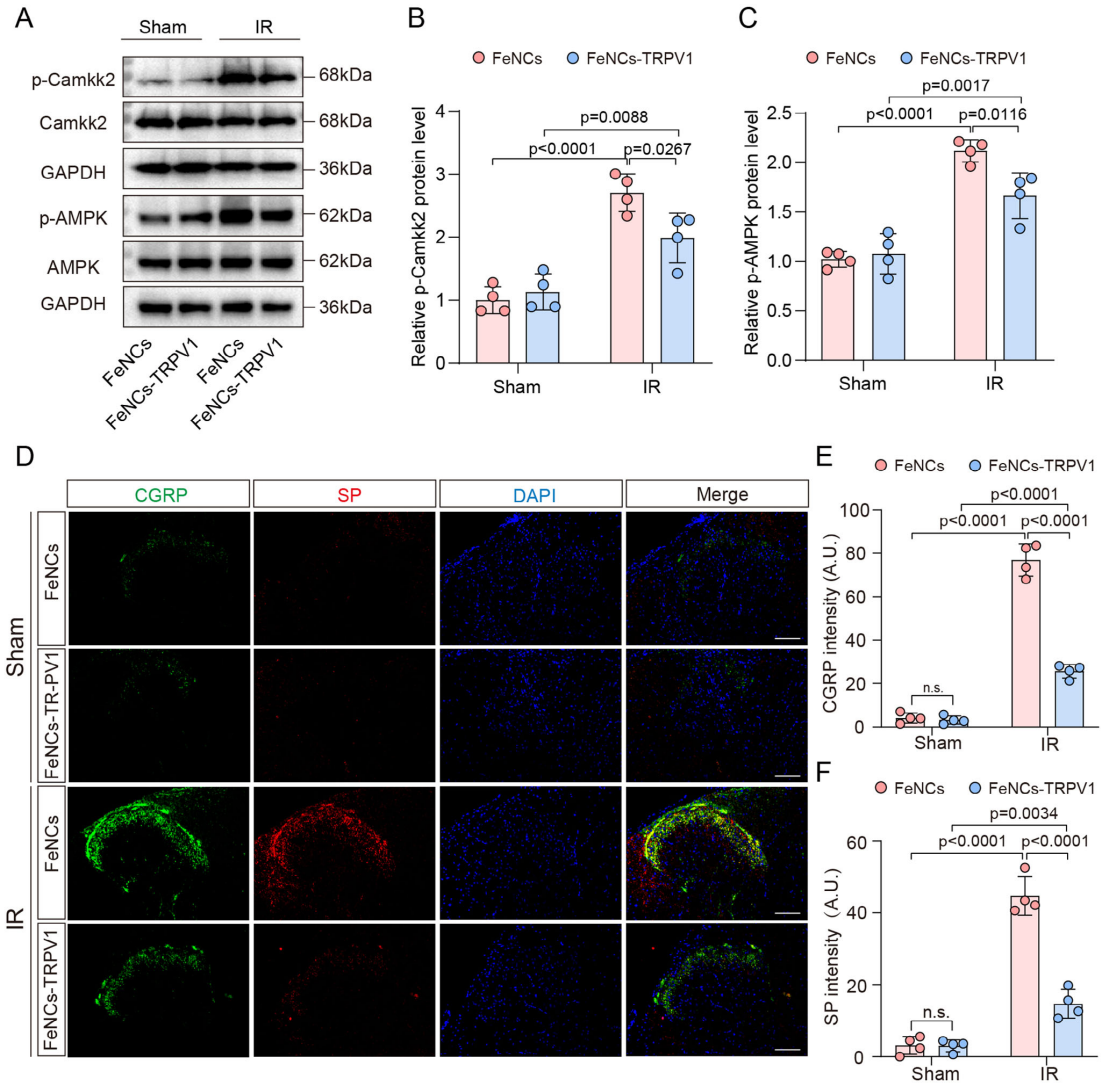
FeNCs‐TRPV1 inhibits calcium‐dependent signaling and neuropeptides release in the spinal cord. A) Representative immunoreactive bands of p‐Camkk2/Camkk2, p‐AMPK/AMPK, and the reference GAPDH in spinal cord tissue samples. B,C) The relative levels of p‐Camkk2 and p‐AMPK were expressed as ratios of their phosphorylated forms to total protein, following normalization to GAPDH, and the value in the FeNCs‐injected Sham group was assigned as 1. D) Representative images showing CGRP and SP immunostaining in the spinal cord issues from rats. Scale bar = 50 µm. E,F) Quantification of CGRP (E) and SP (F) fluorescence intensity (A.U.). Data represent the mean ± SD. Statistical analysis was performed using two‐way ANOVA followed by Tukey's test (*n* = 4 per group).

TRPV1 activation induces channel opening and subsequent Ca^2+^‐dependent downstream signaling cascades. Our findings revealed pronounced upregulation of spinal cord Camkk2 and AMPK phosphorylation levels in the IR group relative to Sham controls (Figure S11A, Supporting Information), indicative of TRPV1 channel activation and consequent neuronal calcium influx during myocardial IR injury. Intriguingly, when exposed to three cycles of ACMF before myocardial ischemia, FeNCs‐TRPV1 effectively suppressed the phosphorylation of Camkk2 and AMPK that was induced by myocardial IR injury. In rats injected with FeNCs‐TRPV1, the protein levels of p‐Camkk2 and p‐AMPK were significantly reduced by 26.6% and 21.4%, respectively, in comparison to rats injected with FeNCs (*p* = 0.0267 and *p* = 0.0116, respectively, **Figure** **6**A–C). Again, the exposure of ACMF to rats without nanocubes injection did not result in discernible effects on the phosphorylation levels of Camkk2 and AMPK (Figure S11A–C, Supporting Information). Additionally, the IR‐induced upregulation in the protein levels of p‐Camkk2 and p‐AMPK in the spinal cord were suppressed by IPC (Figure S11A–C, Supporting Information).

The activation of TRPV1‐positive cardiac sensory neurons induces the release of neuropeptides, such as SP and CGRP, into the dorsal horns of the spinal cord. During myocardial IR injury, the release of these neuropeptides is augmented, which contributes to the increased cardiac sympathetic efferent activity and subsequent myocardial injury.^[^ ^14^ ^]^ When rats were exposed to ACMF prior to myocardial ischemia, the immunostaining intensities of SP and CGRP in the spinal cord were decreased by 67.1% and 66.7%, respectively, as compared to those of rats injected with FeNCs (both *p* <0.0001, Figure 6D–F). In contrast, sham rats injected with FeNCs or FeNCs‐TRPV1 exhibited weak immunostaining intensities of SP and CGRP in the spinal cord (Figure 6D–F). However, in rats without intraspinal injection of nanocubes, ACMF exposure showed no significant effects on the immunostaining intensities of SP and CGRP (Figure S11D–F, Supporting Information). Furthermore, the increased immunostaining intensities of SP and CGRP in the dorsal horns of the spinal cord were significantly suppressed by IPC (Figure S11D–F, Supporting Information). These results demonstrate that repetitive ACMF exposure to rats injected with FeNCs‐TRPV1 can inhibit the activation of the TRPV1 channel during myocardial IR, thereby inhibiting the release of SP and CGRP in the spinal cord, and consequently mitigating cardiac injury.

### 2.7 FeNCs‐TRPV1 Mediates Spinal TRPV1 Channel Desensitization Upon Repeated ACMF Exposure

**Figure 7:**
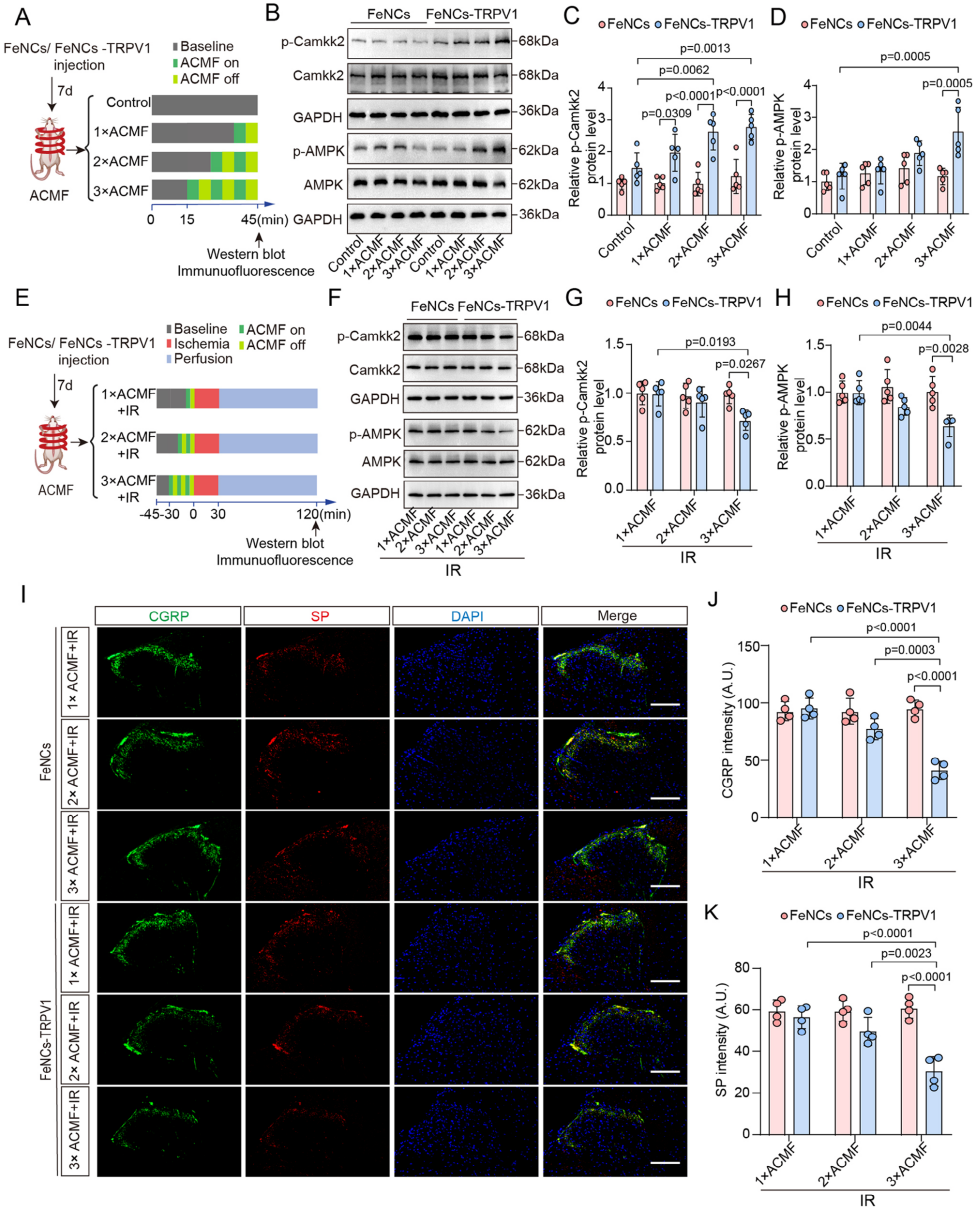
FeNCs‐TRPV1 mediates spinal TRPV1 channel desensitization upon repeated ACMF exposure. A) Schematic diagram of the experimental procedure for 1, 2, or 3 cycles of ACMF exposure without subsequent myocardial IR. B) Representative immunoreactive bands of p‐AMPK/AMPK, p‐Camkk2/Camkk2, and the reference GAPDH in spinal cord tissue samples. C,D) The relative levels of p‐Camkk2 and p‐AMPK were expressed as ratios of their phosphorylated forms to total protein, following normalization to GAPDH, and the value in the FeNCs‐injected Control group was assigned as 1 (*n* = 5 per group). E) Schematic diagram of the experimental procedure for 1, 2, or 3 cycles of ACMF exposure with subsequent myocardial IR. F) Representative immunoreactive bands of p‐Camkk2/Camkk2, p‐AMPK/AMPK, and the reference GAPDH in spinal cord tissue samples. G,H) The relative levels of p‐Camkk2 and p‐AMPK were expressed as ratios of their phosphorylated forms to total protein, following normalization to GAPDH, and the value in FeNCs‐injected rats subjected to 1 cycle of ACMF was assigned as 1 (*n* = 5 per group). I) Representative images showing CGRP and SP immunostaining in the spinal cord issues from rats. Scale bar = 50 µm. J,K) Quantification of CGRP (J) and SP (K) fluorescence intensity (A.U.). Data represent the mean ± SD. Statistical analysis was performed using two‐way ANOVA followed by Tukey's test (*n* = 5 per group in B–D and F–H, *n* = 4 per group in I–K).

To explore the mechanisms underlying FeNCs‐TRPV1‐mediated cardioprotection, we next examined the effects of different ACMF exposure cycles on spinal TRPV1 channel activation. Rats injected with FeNCs or FeNCs‐TRPV1 were subjected to one, two, or three cycles of ACMF exposure (5 min on/5 min off), in the absence or presence of subsequent myocardial IR (**Figure** **7**A,E). In the absence of myocardial IR, phosphorylated Camkk2 levels in FeNCs‐TRPV1‐injected rats elevated as ACMF cycles were increased (Figure 7B,C), whereas AMPK phosphorylation was enhanced only following three cycles of ACMF exposure (Figure 7B,D). However, the immunofluorescence intensities for both SP and CGRP were weak, and there were no significant differences between the FeNCs‐TRPV1 and FeNCs groups across different ACMF exposure cycles (Figure S12, Supporting Information). These data indicate that FeNCs‐TRPV1 can induce moderate TRPV1 activation and downstream Ca^2+^ signaling under ACMF exposure, but the stimulus was insufficient to trigger neuropeptide release.

When rats were subjected to three cycles of ACMF exposure followed by myocardial IR injury, phosphorylated Camkk2 and AMPK levels in the FeNCs‐TRPV1 group were both significantly lower than those in the FeNCs group (*p* = 0.0267 and *p* = 0.0028, respectively; Figure 7F–H). In contrast, no significant differences between the two groups were observed after one or two cycles of ACMF exposure. Additionally, immunofluorescence intensities of SP and CGRP were significantly reduced in the FeNCs‐TRPV1 group compared with the FeNCs group only following three cycles of ACMF exposure (Figure 7I–K). Consistently, myocardial IR injury was significantly attenuated in FeNCs‐TRPV1‐injected rats relative to FeNCs‐injected rats after three cycles of ACMF exposure, with no significant difference observed after one or two cycles (Figure S13, Supporting Information). Collectively, these findings suggest that FeNCs‐TRPV1 mediates pre‐activation of spinal TRPV1 channel upon repeated ACMF exposure, which may render TRPV1 channels less sensitive to subsequent prolonged ischemic stimuli.

## 3 Discussion

**Figure 8:**
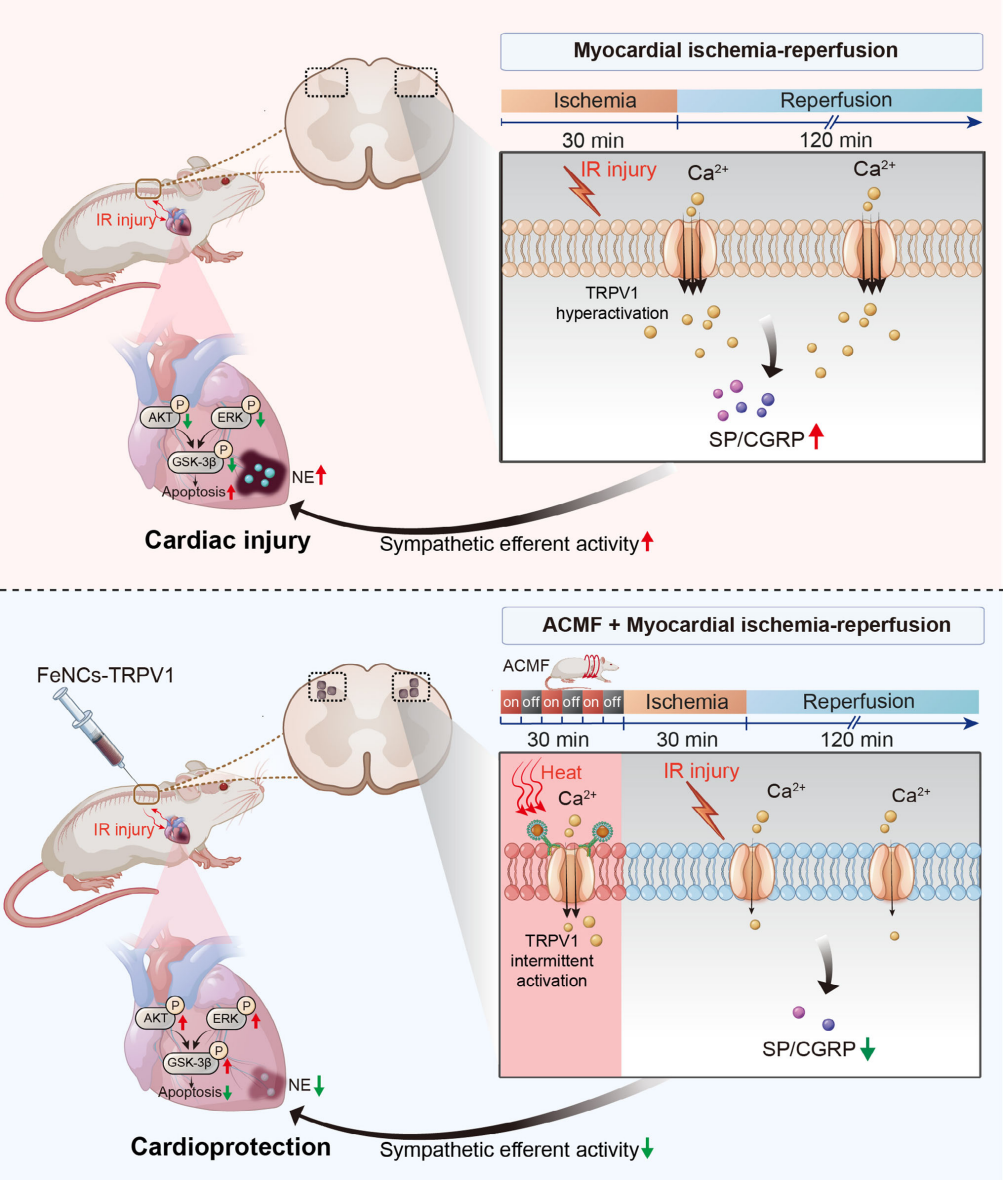
A summary figure illustrating that FeNCs‐TRPV1 exerts cardioprotective effects upon exposure to ACMF through magnetothermal effects. When the FeNCs‐TRPV1 nanocubes were injected into the rat spinal cord, repetitive and transient ACMF exposure induced the intermittent activation of the TRPV1 channel, which induced TRPV1 desensitization and ultimately prevented the hyperactivation of TRPV1 during the subsequent ischemia and reperfusion phases. This resulted in a significant reduction of myocardial ischemic injury in rats, and the cardioprotection is potentially associated with the reduced release of neuropeptides, decreased cardiac sympathetic efferent activity, enhanced intramyocardial prosurvival signaling, and the inhibition of apoptotic signaling. The summary figure was originally created by the authors and professionally refined by a scientific illustration service. The copyright is retained by the authors.

The role of TRPV1 in cardiac ischemic injury has been extensively studied. However, controversy remains regarding whether its activation exerts cardioprotective or detrimental effects on the heart. In the present study, we demonstrate, for the first time, that remote pre‐activation of spinal TRPV1 channel via FeNCs‐TRPV1 upon repeated ACMF exposure induces TRPV1 desensitization and confers cardioprotection against myocardial IR injury. We have developed iron oxide‐based magnetic nanocubes that are conjugated with an extracellular antibody specific to TRPV1. The FeNCs‐TRPV1 nanocubes were designed to target the TRPV1 channel located on the cell surface, and could induce the opening of the channel upon ACMF through magnetothermal effect. When the FeNCs‐TRPV1 nanocubes were injected into the rat spinal cord, repetitive and transient ACMF exposure induced the intermittent activation of the TRPV1 channel, which induced TRPV1 desensitization and ultimately prevented the hyperactivation of TRPV1 during the subsequent ischemia and reperfusion phases. This resulted in a significant reduction of myocardial ischemic injury in rats, and the cardioprotection is potentially associated with the reduced release of neuropeptides, decreased cardiac sympathetic efferent activity, the enhanced intramyocardial prosurvival signaling, and the inhibition of apoptotic signaling (**Figure** **8**).

Conventional strategies such as TRPV1 gene knockout and pharmacological inhibitors have been commonly employed to modulate this channel. However, given the broad physiological functions of TRPV1, these systemic approaches carry the risk of impairing nociceptive responses and disrupting normal thermoregulation.^[^ ^41^ ^]^ The novelty of our work lies in the first‐time application of magnetic nanomaterials to specifically target the TRPV1 channel during myocardial IR injury. This approach allows for specific modulation of the TRPV1 channel in the spinal cord without interfering with its function in other tissues, thereby avoiding the adverse effects associated with systemic TRPV1 inhibition. Furthermore, by leveraging the magnetothermal effect, we achieve precisely and real‐time modulate the TRPV1 channel activation prior to ischemia, which in turn induced TRPV1 desensitization during subsequent myocardial IR and thus mitigates cardiac injury. To our knowledge, this is the first study to apply magnetothermal modulation targeting TRPV1 in the context of myocardial infarction.

Several studies have shown the potential of coupling nanomaterials with TRPV1 antibodies to target the TRPV1 channel.^[^ ^24^, ^26^, ^30^ ^]^ However, most of these antibodies target the intracellular regions of TRPV1. The extracellular antibody utilized in this study is highly specific and targets the third extracellular loop of the rodent TRPV1 channel. This antibody is commercially available and is designed for detecting TRPV1 channels on the cell surface, being particularly suitable for intact living cells.^[^ ^42^ ^]^ Given that the TRPV1 channel typically undergoes translocation to the cell membrane before its activation and opening, the FeNCs‐TRPV1 nanocubes are capable of directly binding TRPV1 channels on the cell surface, without the need to pass through the membrane. We observed that, following incubation, the FeNCs‐TRPV1 nanocubes were predominantly located around the cell surface only in rTRPV1‐transfected F11 cells, indicating the specific binding of the nanocubes to TRPV1 channels located on the cell membrane. In addition, we confirmed through whole‐cell patch‐clamp experiments that the binding of this extracellular antibody to the TRPV1 channel has no effect on capsaicin‐induced inward current, indicating that FeNCs‐TRPV1 can specifically bind the TRPV1 channel without affecting the channel's activity.

Fe‐based nanomaterials possess the property of superparamagnetic, enabling them to be heated rapidly under an ACMF exposure. This heating could be converted into cellular signals by activating the temperature‐sensitive channel to allow Ca^2+^ entry into cells. Our study presents evidence that FeNCs‐TRPV1 can rapidly heat up when exposed to ACMF in a concentration‐ and intensity‐dependent manner. By taking advantage of the magnetothermal property of FeNCs and the heat‐sensitive characteristic of the TRPV1 channel, we demonstrated that FeNCs‐TRPV1 induced Ca^2+^ influx in rTRPV1‐transfected F11 cells and WT primary DRG neurons (with endogenous TRPV1 expression) upon ACMF exposure. The opening of the TRPV1 channel was further verified by the increased phosphorylation levels of Camkk2 and AMPK, the downstream effectors of TRPV1‐mediated Ca^2+^ signaling.^[^ ^26^ ^]^ These in vitro results support the use of FeNCs‐TRPV1 in animal experiments to specifically target the TRPV1 channel and induce the activation and the opening of the channel under ACMF.

The FeNCs‐TRPV1 or the control FeNCs were stereotactically injected into the upper thoracic spinal cord, as the central terminals of cardiac afferent sensory neurons are mainly located between the T2‐T6 segments.^[^ ^12^, ^43^ ^]^ After injected, these nanocubes were predominantly localized in the dorsal horns of the spinal cord, rendering them to specifically bind to TRPV1 channels expressed on the central endings of cardiac sensory neurons. Rats injected with FeNCs‐TRPV1 were exposed to three cycles of transient ACMF (5 min on/5 min off) to induce intermittent activation of spinal TRPV1 channels prior to myocardial ischemia. This resulted in a significant reduction in myocardial infarct size, serum cTnI concentration, as well as arrhythmia scores. The cardioprotective effects might be attributed to the decreased cardiac sympathetic efferent activity, the activation of prosurvival kinases, and the inhibition of apoptotic signaling in the myocardium. The rats injected with FeNCs‐TRPV1 were exposed to ACMF in a preconditioning manner similar to the classic IPC. We found that this ACMF exposure protocol exerted cardioprotective effects comparable to those of IPC. Given that the application of IPC is largely hampered by its invasive nature, such a non‐invasive approach might serve as an alternative strategy for cardioprotection.

In the thoracic spinal cord, myocardial IR‐induced elevation of phosphorylated Camkk2 and AMPK levels was significantly reduced by FeNCs‐TRPV1 under three cycles of ACMF exposure. The underlying mechanisms could be explained by the fact that repeated and transient ACMF exposure induces repetitive intermittent activation of the TRPV1 channel, which may lead to the channel desensitization during the subsequent prolonged ischemia and reperfusion phases. Consequently, I/R‐induced TRPV1 hyperactivation and the activation of downstream Ca^2+^ signaling were suppressed by FeNcs‐TRPV1 under repeated ACMF exposure. The excitation of TRPV1‐positive cardiac afferent sensory neurons induced by myocardial IR injury leads to the release of SP and CGRP in the spinal cord.^[^ ^39^ ^]^ Released SP and CGRP within the spinal cord can function as neurotransmitters on sympathetic nerves, resulting in chest pain and reflex sympathetic excitation.^[^ ^44^ ^]^ We have previously reported that intrathecal administration of antagonists against SP and CGRP in the spinal cord confers protective effects for myocardial IR injury.^[^ ^14^ ^]^ Additionally, IPC‐mediated cardioprotection was found to be associated with reduced release of SP and CGRP in the spinal cord and the consequent inhibition of spinal neuron excitability.^[^ ^45^ ^]^ In this study, we demonstrated that IR‐induced SP and CGRP release in the spinal cord were both reduced in FeNCs‐TRPV1‐injected rats exposed to repeated ACMF. Therefore, the cardioprotection induced by FeNCs‐TRPV1 upon exposure to ACMF might be ascribed to the decreased levels of SP and CGRP within the spinal cord dorsal horns, which, in turn, leads to the reduction of cardiac sympathetic efferent activity, thereby protecting the heart against ischemic injury.

Magnetic fields can penetrate deep into biological tissues without causing cellular damage or energy attenuation.^[^ ^46^ ^]^ This is an advantage over other external stimuli like electromagnetic induction, optical, or acoustic signals, which are largely impeded by tissue absorption or energy scattering.^[^ ^30^ ^]^ Previous investigations have documented the application of the magnetothermal strategy targeting TRPV1 channels under several circumstances, including deep brain stimulation,^[^ ^47^ ^]^ Parkinson's disease,^[^ ^31^ ^]^ osteoarthritis, and adrenal hormones regulation.^[^ ^30^, ^48^ ^]^ Nevertheless, the employment of this approach in the treatment of cardiovascular diseases has yet to be reported. In preclinical studies, neuromodulation therapy such as spinal cord stimulation (SCS) has been employed to mitigate cardiac sympathoexcitation and ventricular arrhythmia during myocardial ischemia and reperfusion.^[^ ^49^, ^50^, ^51^ ^]^ It is worth highlighting that SCS has also been applied in patients for the treatment of heart failure,^[^ ^52^ ^]^ atrial fibrillation, and refractory angina.^[^ ^53^, ^54^ ^]^ However, the invasive implantation of electrodes in SCS poses risks of hardware failure, hemorrhage, and infection.^[^ ^31^ ^]^ FeNCs‐TRPV1 mediated activation of spinal TRPV1 through magnetothermal effect may provide a minimally invasive strategy for cardioprotection in patients with ischemic heart diseases.

In this study, FeNCs‐TRPV1 was precisely delivered to the spinal cord through stereotactic injection. Despite the invasive nature of this method, we observed no motor or sensory dysfunction of rats after injection. In clinical practice, image‐guided epidural spinal injection has been commonly performed in patients with spinal pain, showing great accuracy and safety.^[^ ^55^ ^]^ We speculate that such an injection strategy could be a possible way for FeNCs‐TRPV1 clinical use in the future. The injected FeNCs‐TRPV1 particles within the spinal cord are not easily metabolized and remain intact for several months after injection. Nevertheless, Fe‐based magnetic materials exhibit minimal cytotoxicity and great biocompatibility.^[^ ^47^ ^]^ Consistently, our in vitro and in vivo data revealed no detectable toxicity of the nanocubes toward mammalian cells or major organ systems. Moreover, the retained FeNCs‐TRPV1 in the spinal cord could be utilized to chronically control spinal TRPV1 channels in vivo. It would be of great significance for the future to investigate the effects of FeNCs‐TRPV1 mediated spinal TRPV1 activation under ACMF in post‐myocardial infarction remodeling and heart failure.

In summary, this study demonstrates that FeNCs conjugated with TRPV1 extracellular antibody are capable of specifically binding to spinal TRPV1 channels and exerting cardioprotective effects upon exposure to repeated ACMF through magnetothermal effect. These findings provide a novel potential therapeutic strategy that might alleviate myocardial IR injury through remotely magnetic modulation of TRPV1 channels within the spinal cord.

## 4 Experimental Section

### Synthesis of FeNCs and FeNCs‐TRPV1

The Fe_3_O_4_‐based nanocubes, i.e., FeNCs were initially synthesized through the high‐temperature decomposition of Fe(Ac_3_) (Ac, acetate), as outlined in prior studies.^[^ ^56^ ^]^ Specifically, 0.7 g ferric acetylacetonate (14024‐18‐1, Aldrich) along with 3.2 mL oleic acid (112‐80‐1, Aladdin) and 0.8 mL oleylamine (1120‐90‐3, Aladdin) were dispersed in 20 mL benzyl ether (103‐50‐4, Thermo Scientific). This mixture was then transferred into a 50‐mL three‐neck round‐bottom flask and heated from room temperature to 220 °C at a constant rate of 3 °C min^−1^ under a nitrogen atmosphere. After maintaining 220 °C for 60 min, the solution gradually transitioned from a brick‐red color to a black suspension. The flask was then additionally heated at the same rate to 290 °C, where it was held for 40 min with ongoing condensation reflux. Upon completion of the reaction and subsequent cooling to room temperature (25 °C), the resulting solid products underwent three washes with absolute ethyl alcohol and underwent separation using an external magnet. Acetone was then introduced to remove any residual dibenzyl ether in the solution, and the nanocrystals were then suspended in chloroform (67‐66‐3, Sinopharm) post‐magnetic separation with two additional washes with acetone (67‐64‐1, Sinopharm).

The biocompatibility and water solubility of the FeNCs are enhanced by attaching PEGylated long‐circulation lipid DSPE‐PEG2000 molecules to their surface.^[^ ^56^ ^]^ To prepare the solution, 80 mg of DSPE‐PEG2000‐COOH solid powder was dissolved in 5 mL of chloroform. Subsequently, 2 mL oleic acid‐coated magnetic manganese‐zinc ferrite nanostructures (5 mg Fe per mL in chloroform) were added. The mixture was then ultrasonicated for 10 min in a 50 mL round‐bottom flask. Following this, 5 mL of deionized water was added, and the mixture was ultrasonicated for 5 min. Then the flask was placed on a rotary evaporator at 70 °C under vacuum. A single layer of DSPE‐PEG2000‐COOH phospholipid molecules was applied to the particle surface. Following cooling and dispersion with an ultrasonic probe for 15 min, the sample was filtered through a 220‐nm pore filter with subsequent centrifugation for ultrafiltration. The resulting clear aqueous nanostructure solution was stored at 4 °C. The surface of the sample was then coated with DSPE‐PEG2000‐COOH phospholipid molecules containing carboxyl groups. The antibody against the extracellular portion of TRPV1 (ACC‐029, Alomone), which features amino groups on its surface, was attached to the FeNCs via an amide bond formed through the EDC/NHS (C_3_H_17_N_3_⋅HCl/C_4_H_5_NO_3_) amide reaction. The unattached antibodies were then filtered out, and the prepared FeNCs‐TRPV1 nanoconjugates were washed several times with deionized water and dispersed in phosphate‐buffered saline (PBS) for further use. The BCA protein assay was used to determine the protein concentration of the antibody‐conjugated FeNCs solutions following the manufacturer's protocol (P0012, Beyotime).^[^ ^25^ ^]^

### Characterization of FeNCs and FeNCs‐TRPV1

The samples of FeNCs and FeNCs‐TRPV1 were evenly dispersed in acetone, and the dispersed nanocubes solution was dripped on the transmission electron microscope grid covered with carbon film to avoid aggregation. And then the solutions were dried in a vacuum in order to ensure the purity and stability of the sample before observation. The morphology of FeNCs and FeNCs‐TRPV1 was analyzed using a TEM (JEM‐2100, JEOL) at 200 kV. Furthermore, zeta potential detection was performed at 25 °C using a Zetasizer Nano‐ZS (Nano‐ZS90, Malvern). In addition, SDS‐PAGE electrophoresis was used to confirm the binding of the antibody on FeNCs. TRPV1 antibody (2 µg), FeNCs (0.4 mg), and FeNCs‐TRPV1 (0.4 mg) were heated at 95 °C for 5 min in denaturing non‐reducing protein loading buffer (P0016P‐2 mL, Beyotime) to denature the protein. Subsequently, all samples were subjected to SDS‐PAGE (10% acrylamide), and the separated proteins were stained with Coomassie brilliant blue (P0017F, Beyotime).

### Magnetothermal Effect of FeNCs‐TRPV1

A custom‐designed ACMF system was employed to perform a magnetothermal heating experiment on a sample under a magnetic field. The magnetothermal effect of FeNCs‐TRPV1 was examined by suspending different concentrations of FeNCs‐TRPV1 (ranging from 0 to 0.4 mg mL^−1^) in PBS and exposing them to ACMF of different intensities (5, 10, and 15 A current voltage). To initiate the experiment, 600 µL of a FeNCs‐TRPV1 sample was carefully positioned at the center of the magnetic induction coil. The temperature‐measuring optical fiber was carefully inserted directly into the sample to avoid interference from the container walls. The ACMF was then activated. An optical fiber thermometer was utilized to accurately monitor the temperature changes within the sample during the heating process. Furthermore, the thermal images were recorded by an infrared thermal imaging camera (Fotric) and processed using AnalyzIR Venus (V6.1.11.491) software. The determined parameters of the alternating magnetic field were as follows: frequency, 1375 kHz; current, 15 A; magnetic field intensity, 0.64 kA m^−1^; magnetic induction coil diameter, 3 cm.

### Cell Culture

Contamination‐free F11 cells (08062601, ECACC, RRID: CVCL_H605) were cultured in DMEM (11965092, Gibco) supplemented with 10% fetal bovine serum (FBS) and 1% penicillin‐streptomycin (C0222, Beyotime) at 37 °C. To maintain the contractile phenotype, cells from generations 3 through 10 were utilized in each experiment. Because F11 cells do not endogenously express the TRPV1 gene, an encoding rat TRPV1 clone (rTRPV1) carried by a pCMV6‐entry vector (NM_031982, Origene) was employed. The rTRPV1 plasmid or the empty vector (PS10001, Origene) was transfected into F11 cells using Lipofectamine 2000 (11668030, Invitrogen). The successful transfection was verified by detecting Myc‐tagged TRPV1 using western blotting and immunofluorescence staining. And the antibodies used in the experiment were rabbit anti‐TRPV1 (4258, Abcam) and rabbit anti‐Myc‐tag (2278, CST).

### Cell Viability Measurement

F11 cells transfected with the rTRPV1 plasmid or the vector were seeded at a density of 1 × 10^3^ cells per well in a 96‐well plate and cultured overnight. Subsequently, fresh medium supplemented with filter‐sterilized FeNCs or FeNCs‐TRPV1 at different concentrations of 0.01, 0.05, and 0.1 mg mL^−1^ were added to each well and incubated for 24 h. Additionally, fresh medium containing 0.1 mg mL^−1^ FeNCs or FeNCs‐TRPV1 was added to the wells and incubated for 24, 48, and 72 h. Cell viability was assessed using the CCK‐8 (BL001A, Biomiky) assay, as previously reported.^[^ ^57^ ^]^ After incubation of the cells with FeNCs or FeNCs‐TRPV1, 10 µL CCK‐8 solution (5 mg mL^−1^) was added to each well followed by an additional 1.5 h incubation at 37 °C. The absorbance at 450 nm was then measured using a microplate reader (RT 6000, Rayto). Cells treated with PBS served as the control. Additionally, cell‐free wells containing FeNCs or FeNCs‐TRPV1 served as blank controls, and their absorbance values were subtracted during data analysis.

### Detection of Iron Nanocubes in F11 Cells

F11 cells transfected with rTRPV1 or vector were incubated with FeNCs‐TRPV1 or FeNCs at the iron concentration of 0.1 mg mL^−1^ for 2 h. Cells were then detached, washed with PBS, and pelleted by centrifugation. The pellet was then fixed with 2.5% glutaraldehyde (abs9277, Absin), and sectioned for TEM examination to observe the localization of the nanocubes in F11 cells.

In addition, the cells incubated with FeNCs‐TRPV1 or FeNCs mentioned above were fixed with 4% paraformaldehyde for 15 min. And the fixed cells were then treated with Prussian blue staining reagent (G1424, Solarbio) at 37 °C for 30 min to stain the magnetic nanocubes. Subsequently, the reaction was stopped with pure water, and cells were counterstained with eosin. After washing with PBS, cells were examined under a microscope to ascertain the localization of nanocubes.

### Whole‐Cell Patch‐Clamp Recording

F11 cells successfully transfected with the rTRPV1 plasmid were cultured for 48 h, then divided into 3 groups and incubated with extracellular TRPV1 antibody (TRPV1 Ab, ACC‐029, Alomone), FeNCs‐TRPV1, or PBS for 2 h. Meanwhile, cells transfected with the empty vector were set as the control group. After incubation, all cells were washed 3 times with MEM medium. Subsequently, the cells were treated with capsaicin (1 µmol L^−1^) for 30 sec, and then whole‐cell patch clamp recordings were made under visual guidance on F11 cells using pipettes (3–5 MΩ) filled with intracellular solution, with a holding potential of ‐70 mV. The pipette (intracellular) solution contained (m_M_): 140 KCl, 2 MgCl_2_, 10 EGTA, 10 HEPES, 5 Mg‐ATP, pH 7.4 with KOH. The extracellular solution contained (m_M_): 138 NaCl, 2 CaCl_2_, 5.4 KCl, 1 MgCl_2_, 10 glucose, and 10 HEPES, pH 7.4 with NaOH. Physiological data were analyzed using Clampfit 10.7 software (Molecular Devices, USA).

### Animals

All animals used in this study were housed in the Animal Experiment Center of Anhui Medical University under standard conditions (12‐h light‐dark cycle) and ad libitum access to food and water. The animal's living conditions were meticulously monitored, with a temperature range of 21 ± 2 °C and humidity maintained at 55 ± 5%, to promote their well‐being and minimize potential stress factors that could impact the study outcomes. All animal studies were conducted in accordance with the guidelines outlined in the National Institutes of Health Guide for the Care and Use of Laboratory Animals (NIH Publication No. 85‐23, revised in 1996) and the ARRIVE guidelines (Animal Research: Reporting of In Vivo Experiments). All animal experiments were approved by the Institution Animal Care and Use Committee of Anhui Medical University (approval number: LLSC20232227). Detailed information regarding the number of animals allocated in experimental groups is presented in Table S2 (Supporting Information).

### Isolation of Adult Mouse Primary DRG Neurons

DRG neurons were cultured following established protocol.^[^ ^2^ ^]^ Adult WT and TRPV1^−/−^ were euthanized by cervical dislocation under sevoflurane anesthesia. The DRGs were aseptically extracted and collected in Hanks' balanced salt solution (HBSS, C0219, Beyotime) and then digested with collagenase IA (5 mg mL^−1^, C9891, Sigma) in HBSS at 37 °C for 45 min and then further digested with trypsin (1 mg mL^−1^, T4799, Sigma) in HBSS at 37 °C for 15 min. The isolated cells were cultured in medium containing 10% FBS (heat‐inactivated) and DMEM supplemented with penicillin‐streptomycin. After isolation, cells were seeded on glass coverslips coated with poly‐L‐lysine (20 µg mL^−1^, P1399, Sigma) and laminin (20 µg mL^−1^, L2020, Sigma) and allowed to grow for 2 days before Ca^2+^ imaging experiments. The primary DRG neurons were identified by immunofluorescence staining of MAP2 (a neuron marker) and TRPV1. The primary antibodies used were mouse anti‐MAP2 (ab254143, Abcam) and rabbit anti‐TRPV1 (4258, Abcam), as well as the second antibodies conjugated with Alexa Fluor 568 or Alexa Fluor 488 (Invitrogen).

### Measurement of Ca^2+^ Influx Under ACMF Exposure

To evaluate intracellular Ca^2+^ levels, F11 cells and primary DRG neurons were cultured in glass‐bottom dishes (BS‐15‐GJM, Biosharp) at a density of 1 × 10^4^ cells per dish overnight. Sterilized FeNCs and FeNCs‐TRPV1 (0.1 mg mL^−1^) were then added to each well along with fresh culture medium and incubated for 2 h. Subsequently, the cells were washed with HBSS and incubated with the Ca^2+^ indicator Fluo‐4 AM (10 µ_M_, BB‐48113, BestBio) for 30 min at 37 °C in the dark. Following this, the cells were exposed to an ACMF with a magnetic field current of 15 A for 5 min.^[^ ^38^ ^]^ After a 5‐min stabilization period after the treatment, fluorescence images of the cells were captured using a confocal laser scanning microscope (LSM900, Zeiss) under 488 nm excitation through a 20× objective lens.

### Western Blot Analysis

Western blot was used to detect the phosphorylation levels of the Camkk2 and the AMPK, and to evaluate the activation of the downstream signal pathway triggered by Ca^2+^ influx. F11 cells transfected rTRPV1 or vector were incubated with FeNCs‐TRPV1 or FeNCs and subsequently exposed to an ACMF as described above. The treated cells were then harvested, and total protein was extracted. Each protein sample (25 µg) was subjected to SDS‐PAGE (10% acrylamide), and transferred onto a polyvinylidene difluoride membrane (IPFL00010, Millipore). The membranes were blocked and incubated with primary antibodies as follows: rabbit anti‐AMPK (5831, CST), rabbit anti‐phospho‐AMPK (Thr172, 2535, CST), rabbit anti‐Camkk2 (DF4793, Affinity), rabbit anti‐phospho‐Camkk2 (Ser511, 12818, CST), and rabbit anti‐GAPDH (ab181602, Abcam). After that, each membrane was incubated with the appropriate horseradish peroxidase‐conjugated anti‐rabbit IgG secondary antibody (C31460100, Thermo Fisher Scientific). The blots were detected using an enhanced chemiluminescence detection kit (34095, Thermo Fisher Scientific) by a Gel Imager (UVP ChemStudio 515). The protein levels were analyzed utilizing Image J software (NIH) by an independent observer, who was blinded to the experimental conditions. The relative phosphorylated protein levels were expressed as ratios of their phosphorylated forms to total protein, following normalization to GAPDH.

### Intra‐Spinal Microinjection of FeNCs‐TRPV1

Adult male Sprague‐Dawley rats (body weight 240–260 g) were anesthetized with Zoletil 50 (40 mg kg^−1^, 1:1 tiletamine/zolazepam mixture, Virbac) via intramuscular (i.m.) injection and then positioned on a stereotaxic apparatus (RWD). The thoracic spinal cord was exposed, and FeNCs‐TRPV1 or FeNCs solution was bilaterally administered into the segments T2‐T3 using a 10 µL glass micropipette and a Micro Syringe Pump Controller (WPI).^[^ ^14^ ^]^ Each 250 ng of FeNCs‐TRPV1 or FeNCs with a volume of 250 nL were bilaterally injected into the spinal dorsal horn at a depth of 200 µm and at a rate of 200 nL min^−1^. The micropipette was left in place within the spinal cord for an additional 5 min before removal to ensure complete absorption. After the injection, the midline muscles and skin wound were closed via a suture. The rats were provided with subcutaneous buprenorphine (Y01235B, Slsbio) for postoperative pain relief and allowed to recover for 7 days before further experimentation.

### Detection of Iron Nanocubes within the Spinal Cord

Seven days after intraspinal injection of nanocubes, in situ T_2_ and T_2_^*^ MRI scans were conducted to determine the localization of the magnetic nanocubes. Initially, the rats were anesthetized with an injection (i.m.) of Zoletil 50 and positioned in a 3.0 T MRI system (Siemens) using a rat coil and stent for in vivo MRI, while maintaining a body temperature of ≈ 37 °C. The MRI scan sequence included T_2_, T_2_^*^, and diffusion‐weighted imaging with specific parameters: TR/TE = 408/3.5 ms, flip angle = 30°, field of view = 27 mm × 27 mm, slice thickness = 1 mm, and matrix = 256 × 256. Cross‐sectional imaging was performed to visualize the spinal cord.

Subsequently, the rats were euthanized, and PBS was perfused through the aorta to remove all blood from the circulatory system. The spinal cord segment between T2‐T3 was then fixed in 4% paraformaldehyde at room temperature for at least 24 h. After dehydration with ethanol, the spinal cord was embedded in paraffin, longitudinally sectioned into 10 µm slices using an RM2016 microtome (Leica), stained with Prussian blue (G1424, Solarbio) for iron detection, and analyzed using a slide scanner (VS200, Olympus).

### Establishment of Rat Myocardial IR Injury Model

Rats were anesthetized with Zoletil 50 (40 mg kg^−1^, i.m.) and underwent tracheotomy and tracheal intubation. Mechanical ventilation with room air was provided using an apparatus rodent respirator (RWD) at a rate of 70–80 breaths per minute and a tidal volume of 20–30 mg kg^−1^. Body temperature was maintained at 37 ± 1 °C with a heating pad. Real‐time electrocardiogram monitoring and arrhythmia scoring were performed using a Power‐Lab monitoring system (AD Instruments). Myocardial IR injury was induced by occluding the left anterior descending coronary artery using a 5‐0 suture for 30 min, followed by releasing the ligation for 120 min to achieve reperfusion. Myocardial ischemia was confirmed by ST‐segment changes in the electrocardiogram (ECG) and cardiac cyanosis. During the procedures of ischemia and reperfusion, the incidence of arrhythmia was detected using ECG, and the arrhythmia score was qualitatively assessed as described before.^[^ ^14^, ^58^ ^]^

At the end of perfusion, the blood samples were taken from the rats and used for measuring serum concentrations of cTnI (OKCD00729, Aviva Systems Biology) and norepinephrine (E‐EL‐0047c, Elabscience) using the enzyme‐linked immunosorbent assay (ELISA) method. To evaluate myocardial infarction, the heart was taken out, and the left coronary artery was reoccluded. 0.5 mL of 0.5% Evans blue dye solution (ST3273, Beyotime) was perfused retrogradely into the aorta and coronary arteries to outline the ischemic risk area. The hearts were then sliced into sections of 1 mm thickness, submerged in a 1% aqueous solution of 2,3,5‐triethylenetetrazolium chloride (TTC, BCCH9847, Sigma), and incubated at 37 °C for 15 min. After staining, the viable myocardium turned red, and the infarcted area turned white. Subsequently, the tissue was fixed in 4% paraformaldehyde. The IS, AAR, LV, and RV areas were then measured using Image J software (NIH). The infarct volume was expressed as the ratio of IS to AAR (IS/AAR). The measurement was conducted by a blind independent investigator.

In another set of experiments, total protein was extracted from heart tissues to detect the protein levels of prosurvival kinases and apoptosis‐related proteins in the myocardium, as described in our recent study.^[^ ^59^ ^]^ Additionally, total protein was extracted from spinal cord tissues to detect the phosphorylation of Camkk2 and AMPK, as described in 4.10. The primary antibodies used in the experiment were as follows: rabbit anti‐Akt (4691, CST), rabbit anti‐phospho‐Akt (Ser473, 4060, CST), rabbit anti‐ERK1/2 (4695, CST), rabbit anti‐phospho‐ERK1/2 (Thr202/Tyr204, 4370, CST), rabbit anti‐GSK‐3β (12456, CST), rabbit anti‐phospho‐GSK‐3β (Ser9, 5558, CST), rabbit anti‐caspase‐3 (9662, CST), rabbit anti‐cleaved caspase‐3 (9661, CST), rabbit anti‐Bcl‐2 (26593‐1‐AP, Proteintech), mouse anti‐Bax (60267‐1‐Ig, Proteintech), rabbit anti‐AMPK (5831, CST), rabbit anti‐phospho‐AMPK (Thr172, 2535, CST), rabbit anti‐Camkk2 (DF4793, Affinity), rabbit anti‐phospho‐Camkk2 (Ser511, 12818, CST) and rabbit anti‐GAPDH (ab181602, Abcam). The relative phosphorylated protein levels were expressed as ratios of their phosphorylated forms to total protein, following normalization to GAPDH.

### TUNEL Staining

Cell apoptosis in cardiac tissues was determined using a TUNEL assay kit (C1088, Beyotime Institute of Biotechnology) following the manufacturer's protocols. In brief, left ventricular (LV) tissues were harvested after 120 min of reperfusion. The specimens were processed into paraffin‐embedded blocks and sliced into 4‐µm‐thick sections. These sections were subsequently incubated with the TUNEL reaction mixture, followed by counterstaining with DAPI, and the nuclei of TUNEL‐positive cells showed green fluorescence. Images were captured using a fluorescence microscope (Vert.A1, Zeiss). Data from each group were derived from 6 rat hearts, and analyses were performed in a blind manner to calculate the percentage of TUNEL‐positive cells to the total number of cells.

### Fluorescence Immunohistochemistry

At the end of reperfusion, intracardiac perfusion was performed using PBS and 4% paraformaldehyde. The thoracic spinal cord was then extracted and fixed in 4% paraformaldehyde for 24 h, followed by dehydration with ethanol. Subsequently, the tissue samples were embedded in paraffin, and 10‐µm sections of the T2‐T3 segment were prepared. These sections underwent deparaffinization followed by soaking in a citrate solution and boiling for 10 min to repair the antigen. After washing with PBS three times for 5 min each, a blocking permeabilization solution was added dropwise and allowed to incubate at room temperature for 60 min. The primary antibodies used were rabbit anti‐CGRP (14959, CST) and guinea pig anti‐SP (GP14110, Neuromics). Following overnight incubation with the primary antibodies at 4 °C, the sections were washed with PBS and then incubated with Alexa Fluor 488‐ or 594‐conjugated secondary antibodies (Invitrogen). Finally, the sections were washed with PBS and mounted on slides using a 4, 6‐diamidino‐based anti‐fade mounting medium with DAPI (C1006, Beyotime) before being subjected to a confocal laser scanning microscope (Zeiss).

### Statistics

The data represent the mean ± standard deviation (SD). Statistical analysis utilized GraphPad Prism 8 (Graphpad, San Diego, CA, USA). Either one‐ or two‐way analysis of variance (ANOVA) was performed, followed by Tukey's test to compare multiple groups. To compare two groups, an unpaired two‐tailed Student's *t*‐test was conducted. Statistically significant results were considered when *p* <0.05.

## Conflict of Interest

The authors declare no conflict of interest.

## Author Contributions

X.C., S.L., and Y.Z. contributed equally to this work. S.F.H., Y.Z., and F.Y. conceived and designed the project. X.Y.C., S.Y.L., Y.Z., M.G.Q., K.P., C.W., M.‐Y.D. and L.‐P.N. performed the experimental work. X.Y.C., S.F.H., L.L., N.Y., R.L., and G.Y.Z. analyzed the data. X.Y.C. and S.Y.L. wrote the original draft. S.F.H., Y.Z., F.Y., and G.L.L. revised the article and provided critical feedback. All authors discussed the results.

## Supporting information



Supporting Information

## Data Availability

The data that support the findings of this study are available from the corresponding author upon reasonable request.
