# Supplementary material for: Remote Activation of Spinal TRPV1 by Magnetic Nanocubes Confers Cardioprotection Against Myocardial Ischemia‐Reperfusion Injury
Source: Adv Sci (Weinh). 2025 Dec 12;13(11):e20852. doi: 10.1002/advs.202520852 (PMC12931263; doi:10.1002/advs.202520852)
Supplement: Supplementary file 1 — Supporting Information [file ADVS-13-e20852-s001.doc]

# **Supporting Information**

# **Title**

Remote Activation of Spinal TRPV1 by Magnetic Nanocubes Confers Cardioprotection against Myocardial Ischemia-Reperfusion Injury

**Authors**

*Xueying Cheng^†^; Shuangyu Liu^†^; Yu Zhang^†^; Kang Peng; Muge Qile; Chao Wu; Mengyun Dou; Liu Liu; Na Yang; Rui Liu; Guiyang Zhang; Liangping Ni; Gaolin Liang; Fang Yang^*^; Ye Zhang^*^; Shufang He^*^*

**Supplemental figures**


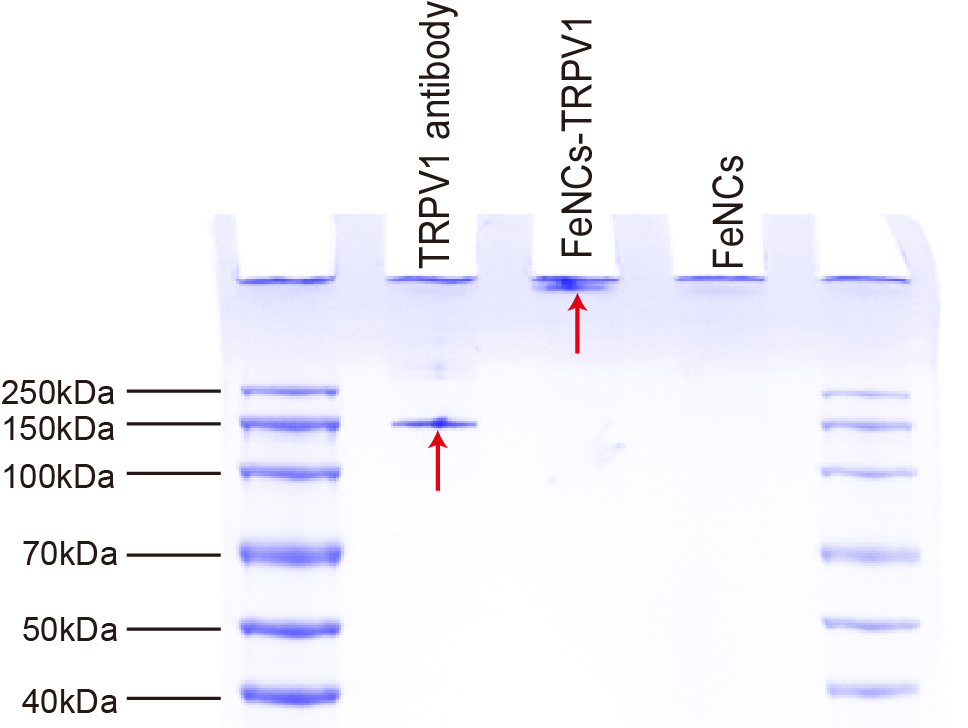


**Figure S1. Coomassie blue staining of an SDS-PAGE gel of TRPV1 antibody, FeNCs and FeNCs-TRPV1.** TRPV1 antibody (2 μg), FeNCs-TRPV1 (0.4 mg), or FeNCs (0.4 mg) samples were heated at 95 ℃ for 5 min in denaturing non-reducing protein loading buffer and loaded onto the 10 % SDS-PAGE gel. After electrophoresis, Coomassie blue staining was used to visualize the protein bands. The red arrows indicate the blue-stained band of TRPV1 antibody.


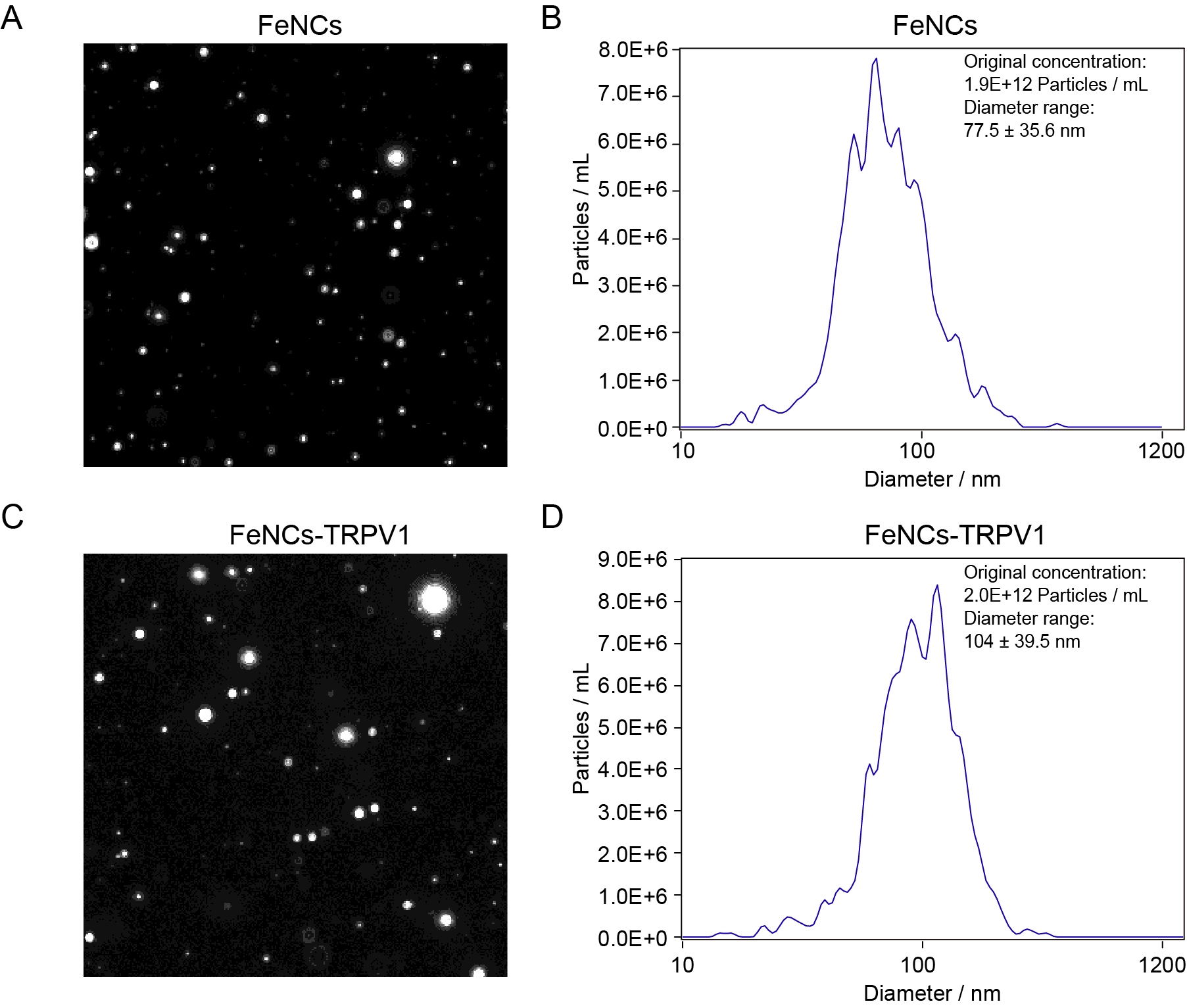


**Figure S2. Particle concentration and size distribution of FeNVs and FeNCs-TRPV1 measured by Nanoparticle Tracking Analysis.** Representative images for particles distribution of FeNCs (A) and FeNCs-TRPV1(C). Peak analysis for FeNCs (B) and FeNCs-TRPV1 (D). Test parameters were set as follow: particle size detection range, 1-10000 nm; laser wavelength, 488 nm; detector gain, 80; shutter speed, 150 ms. Data were automatically analyzed by Software ZetaViewPMX230.


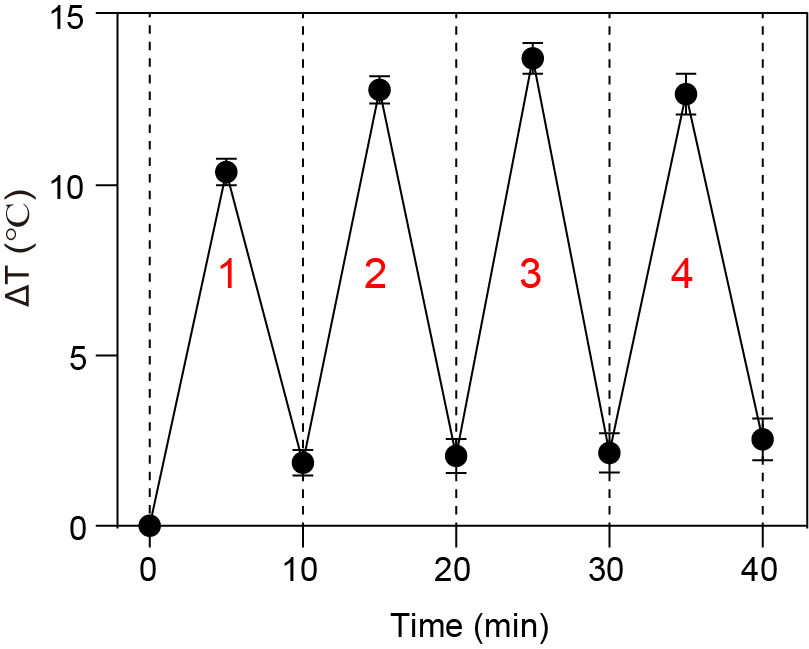


**Figure S3. Transient thermal measurements of FeNCs-TRPV1 (0.1 mg mL^-1^) under repeated on/off cycles of the ACMF (15 A).** Each cycle consisted of a 5 min exposure to ACMF followed by a 5 min cooling phase in a water bath maintained at 27 ℃. Data are shown as mean ± SD of three independent experiments.


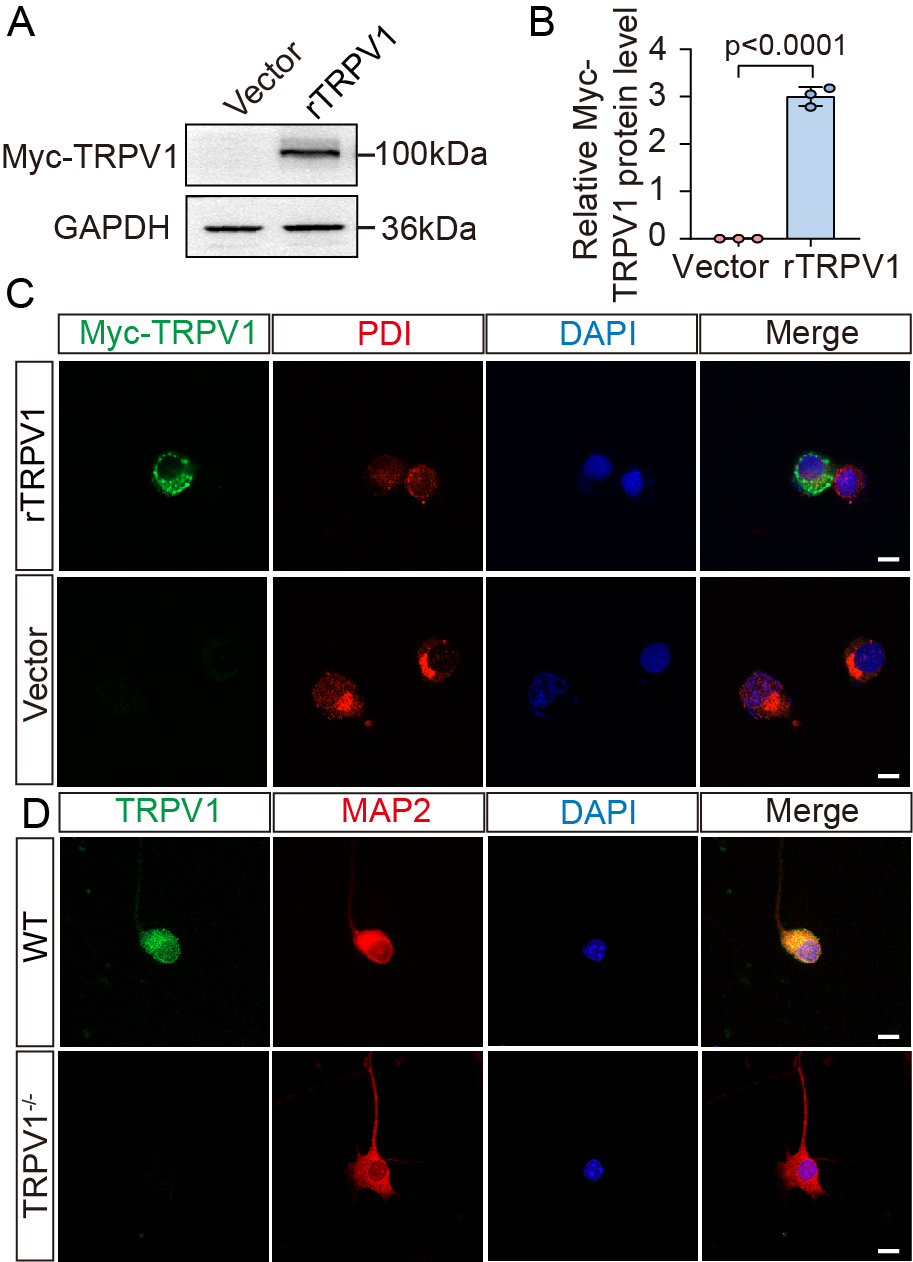


**Figure S4. Detection of TRPV1 expression in F11 cells and primary DRG neurons.** (A) Representative immunoreactive bands of Myc-tagged TRPV1 (Myc-TRPV1) in Vector or rTRPV1-transfected F11 cells. (B) The TRPV1 protein level was normalized to GAPDH. Data represent the mean ± SD. Statistical analysis was performed using unpaired two-tailed t test. (C) Immunofluorescence images of Myc-TRPV1 and protein disulfide isomerase (PDI, an endoplasmic reticulum marker ) in F11 cells. (D) Immunofluorescence images of TRPV1 and microtubule-associated protein 2 (MAP-2, a neuronal marker) in primary　DRG neurons. Scale bar = 10 µm. The antibodies used in the experiment were rabbit anti-Myc-tag (2278, CST), mouse anti-PDI (66422-1-Ig, Proteintech), mouse anti-MAP2 (ab254143, Abcam).


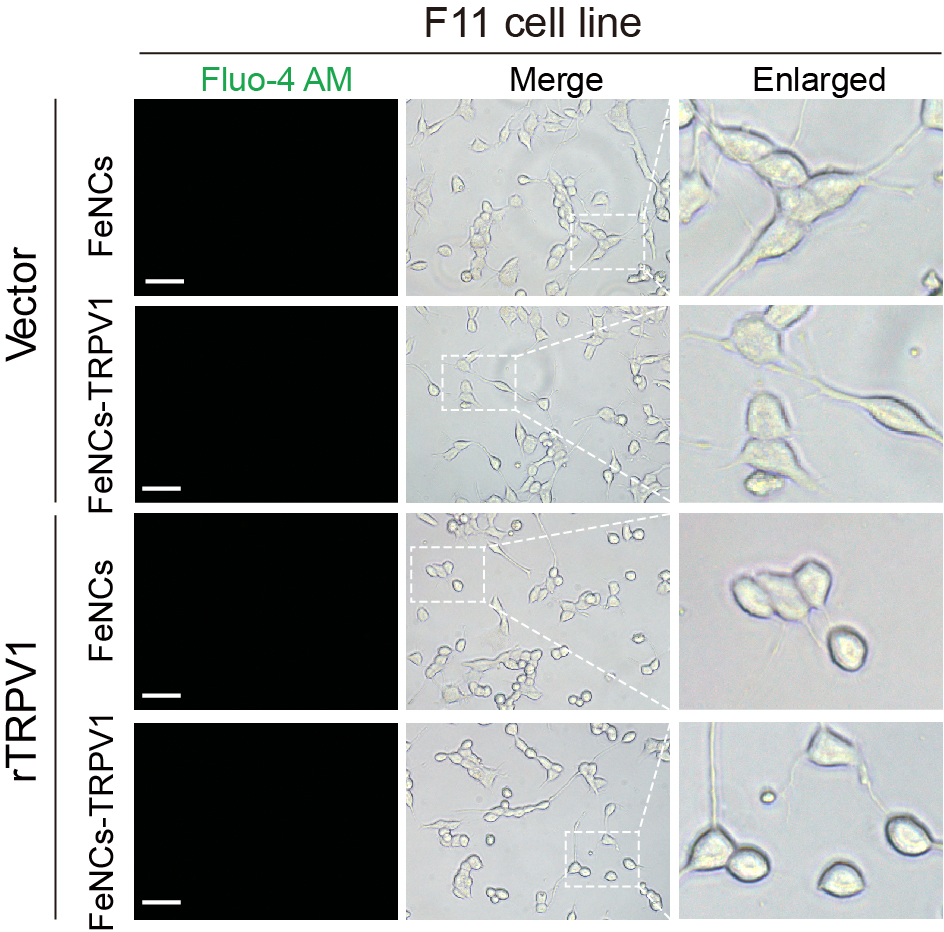


**Figure S5. Effects of FeNCs or FeNCs-TRPV1 on Ca^2+^ influx in the absence of ACMF exposure.** Representative images for intracellular Ca^2+^ signal detected by Fura-4AM fluorescent probe in F11 cells. Scale bar = 50 µm.


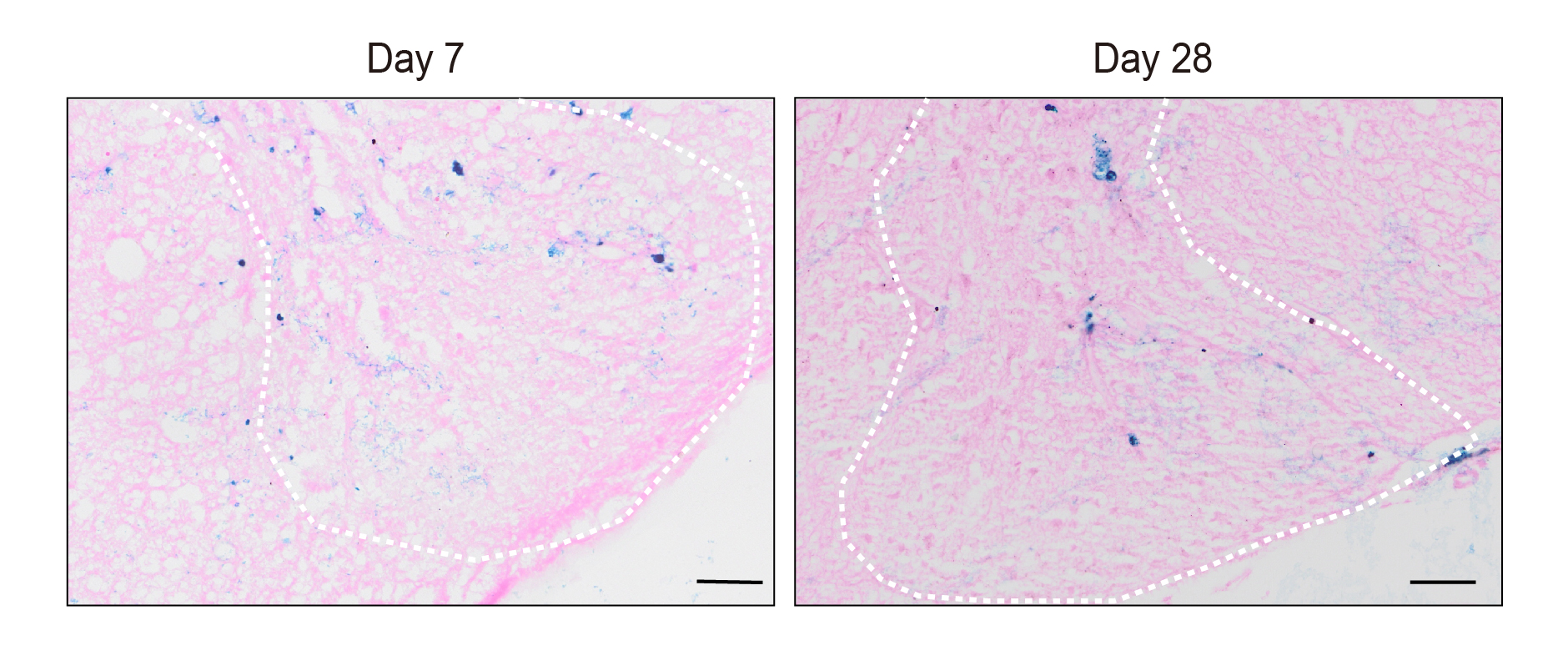


**Figure S6. Prussian blue staining of transverse section of the spinal cord at 7 and 28 days after FeNCs-TRPV1 injection.** The white dotted outline in the figure represents the dorsal horn of the spinal cord. Scale bar = 50 µm.


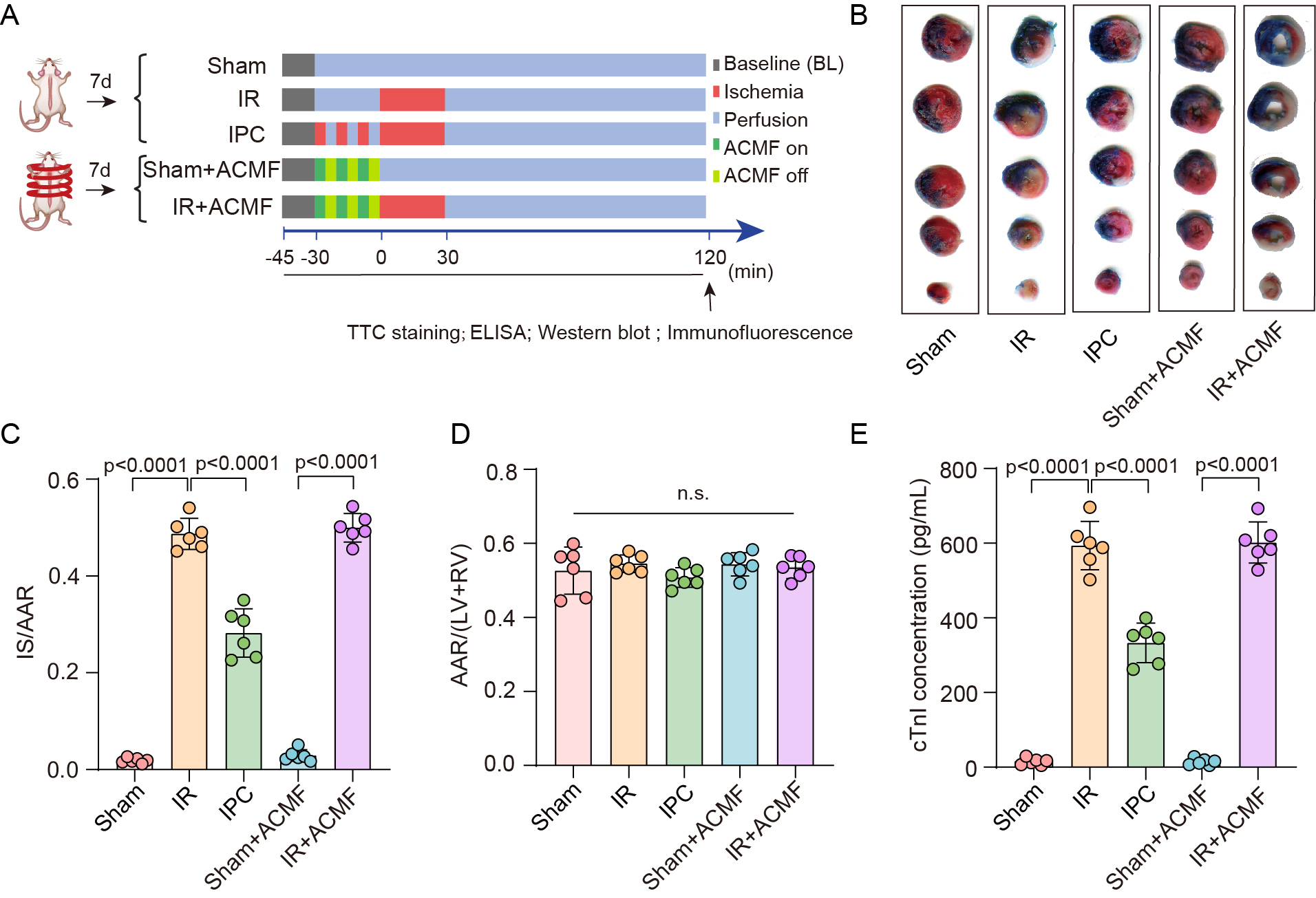


**Figure S7.　Effects of ischemic preconditioning (IPC) on myocardial ischemia-reperfusion injury.** (A) Schematic diagram illustrating the experimental protocols. (B) Representative images of heart sections stained with 2,3,5-triphenyltetrazolium chloride (TTC) and Evans blue. (C) Myocardial infarct volume is expressed as the ratio of infarct size (IS) to area at risk (AAR). (D) The ratio of AAR to total ventricular volume (LV+RV). (E) Serum cardiac troponin I (cTnI) level measured by enzyme-linked immunosorbent assays for each group. Data represent the mean ± SD. Statistical analysis was performed using one-way ANOVA followed by Tukey’s test (*n* = 6 per group). The n.s. represents not significant.


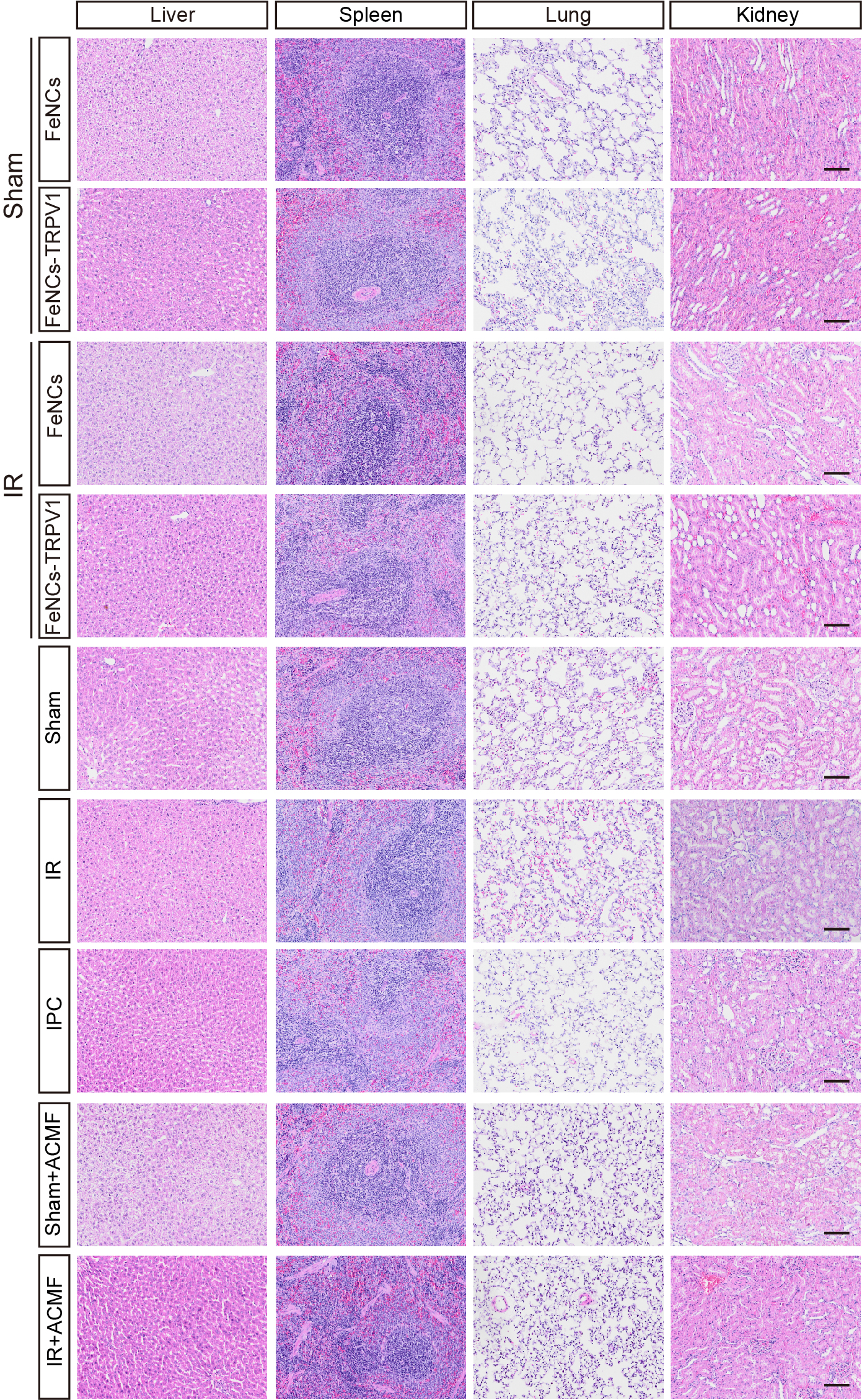


**Figure S8. Safety measurement of nanocubes.** Representative histology (H&E, C0105M, Beyotime) images of major organs (liver, spleen, lung, and kidney) in rats from different groups. No obvious structural or cellular alterations were observed (*n* = 6 per group). Scale bar = 100 µm.


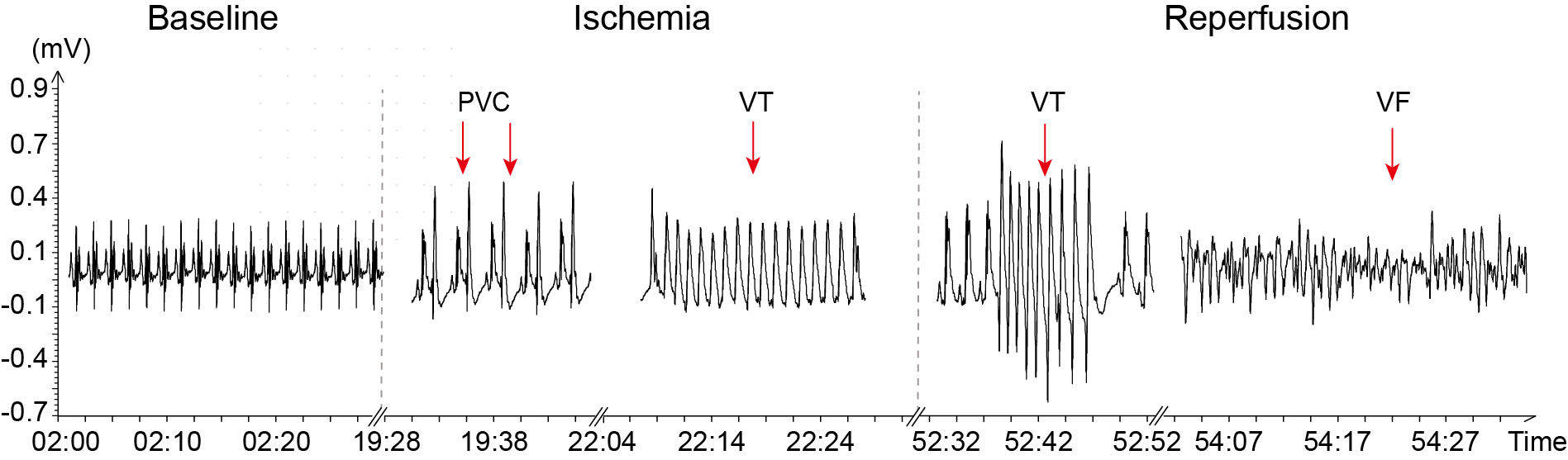


**Figure S9. Representative image for different forms of arrhythmic events during myocardial I/R processes.** PVC, premature ventricular contraction; VT, entricular tachycardia; VF, ventricular fibrillation.


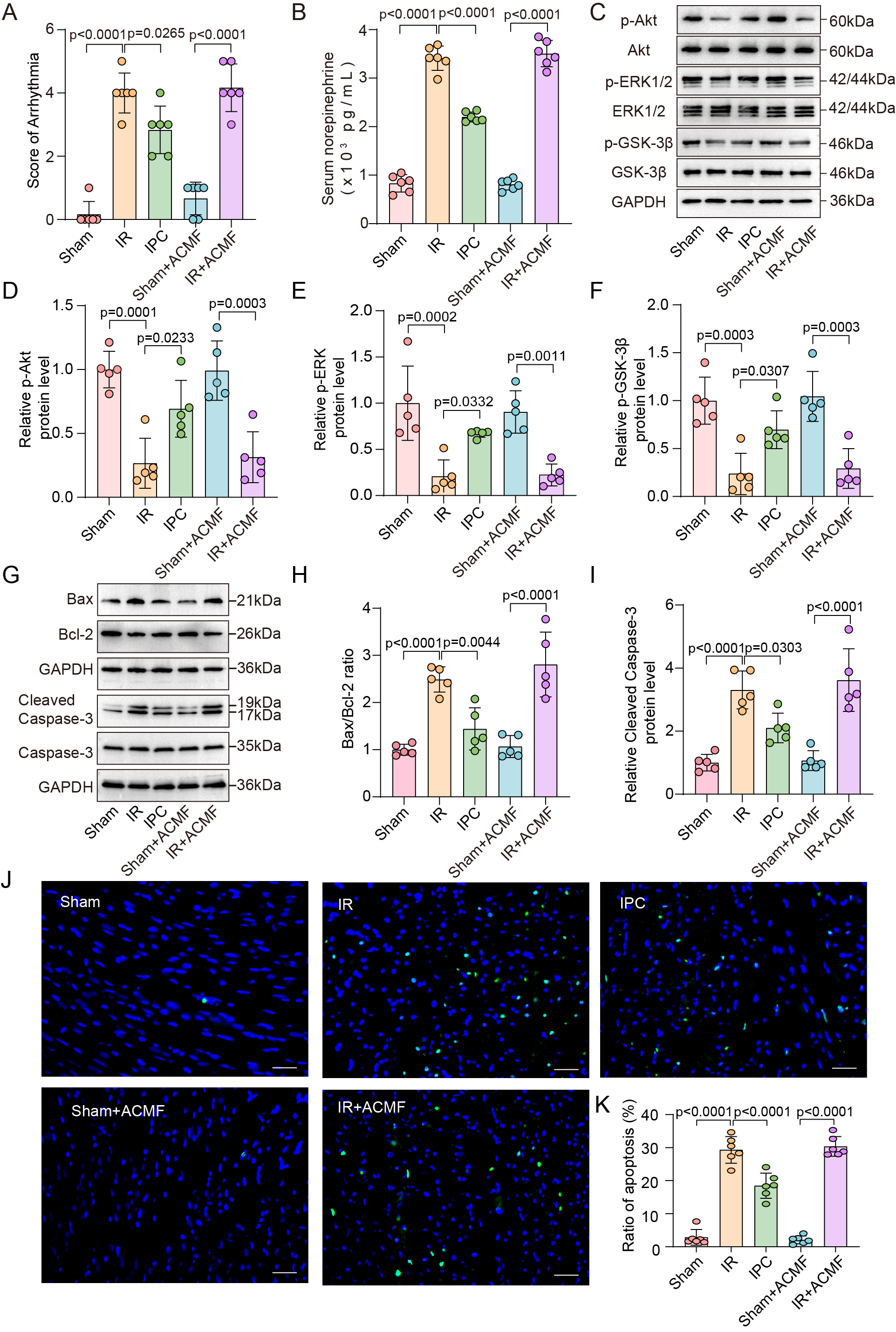


**Figure S10.** **Effects of IPC on arrhythmia, intramyocardial pro-survival signaling and intramyocardial apoptotic signaling.** (A) Arrhythmia score in each group. (B) Serum norepinephrine level measured by enzyme-linked immunosorbent assays. (C) Representative immunoreactive bands of p-Akt/Akt, p-ERK1/2/ERK1/2, p-GSK-3β/GSK-3β, and the reference GAPDH in heart tissue samples. (D-F) The relative levels of p-Akt, p-ERK1/2 and p-GSK-3β were expressed as ratios of their phosphorylated forms to total protein, following normalization to GAPDH, and the value in FeNCs-injected Sham group was assigned as 1. (G) Representative immunoreactive bands of Bax/Bcl-2, cleaved caspase-3/caspase-3, and the reference GAPDH in heart tissue samples. (H, I) The relative ratios of Bax/Bcl-2 and cleaved caspase-3/caspase-3 were calculated following normalization to GAPDH, the value in FeNCs-injected Sham group was assigned as 1 (*n* = 5 per group). (J) Representative images for TUNEL staining in the left ventricular tissues. Scale bar = 20 µm. (K) The number of TUNEL-positive apoptotic cells was quantified and expressed as a percentage of the total number of cells (*n* = 6 rats per group). Scale bar = 20 µm. Data represent the mean ± SD. Statistical analysis was performed using one-way ANOVA followed by Tukey’s test (*n* = 6 per group in A, B; *n* = 5 per group in C-I; *n* = 6 per group in J, K).


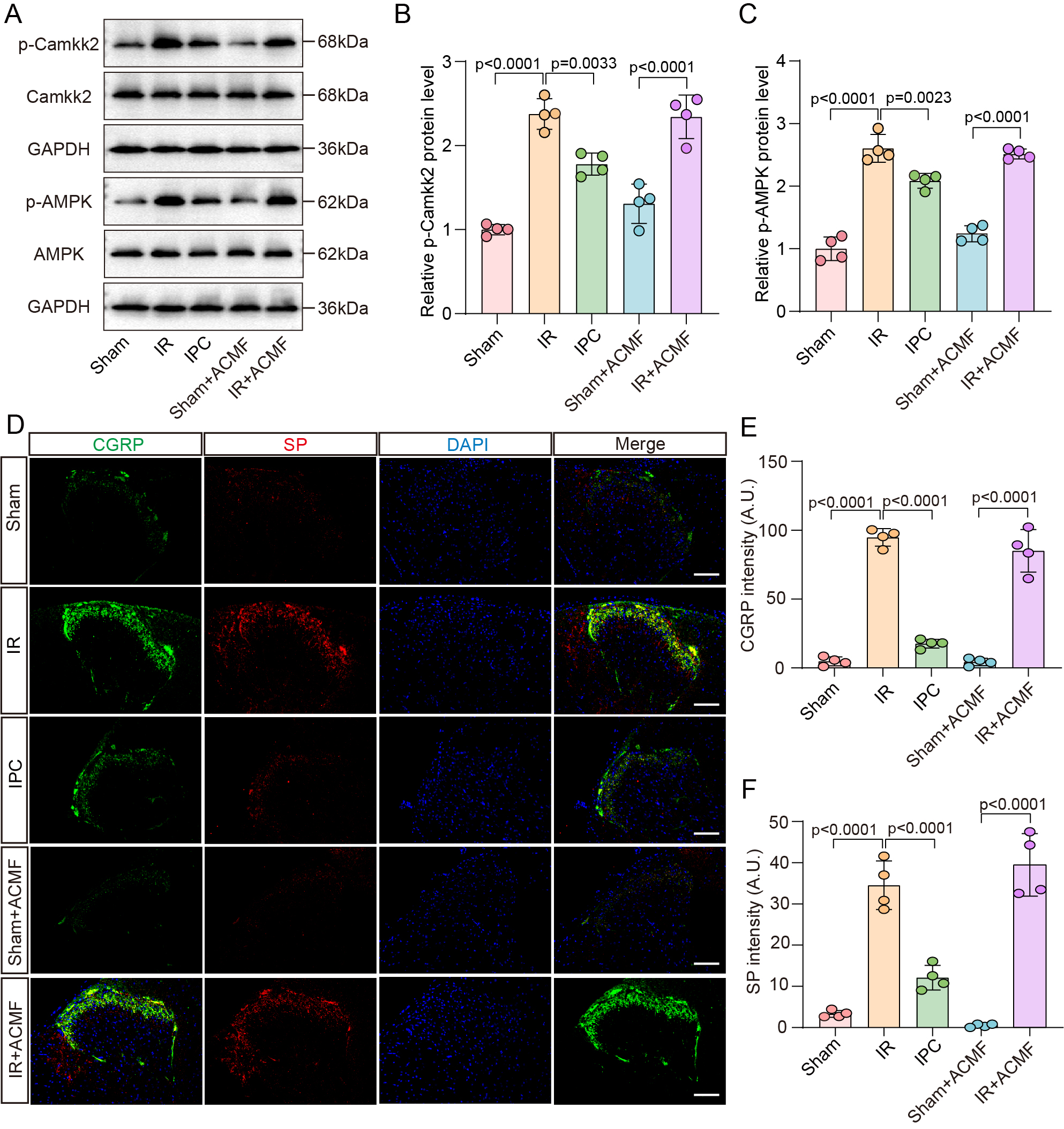


**Figure S11. Effects of IPC on TRPV1 activation and neuropeptide release in spinal cord.** (A) Representative immunoreactive bands of p-AMPK/AMPK, p-Camkk2/Camkk2, and the reference GAPDH in spinal cord tissue samples. (B, C) The relative levels of p-Camkk2 and p-AMPK were expressed as ratios of their phosphorylated forms to total protein, following normalization to GAPDH, and the value in FeNCs-injected Sham group was assigned as 1 (*n* = 4 per group). (D) Representative images showing CGRP and SP immunostaining in spinal cord. Scale bar = 50 µm. (E, F) Quantification of CGRP (E) and SP (F) fluorescence intensity (arbitrary units, A.U.). Data are presented as the mean ± SD. Statistical analysis were performed using one-way ANOVA followed by Tukey’s test (*n* = 4 per group).


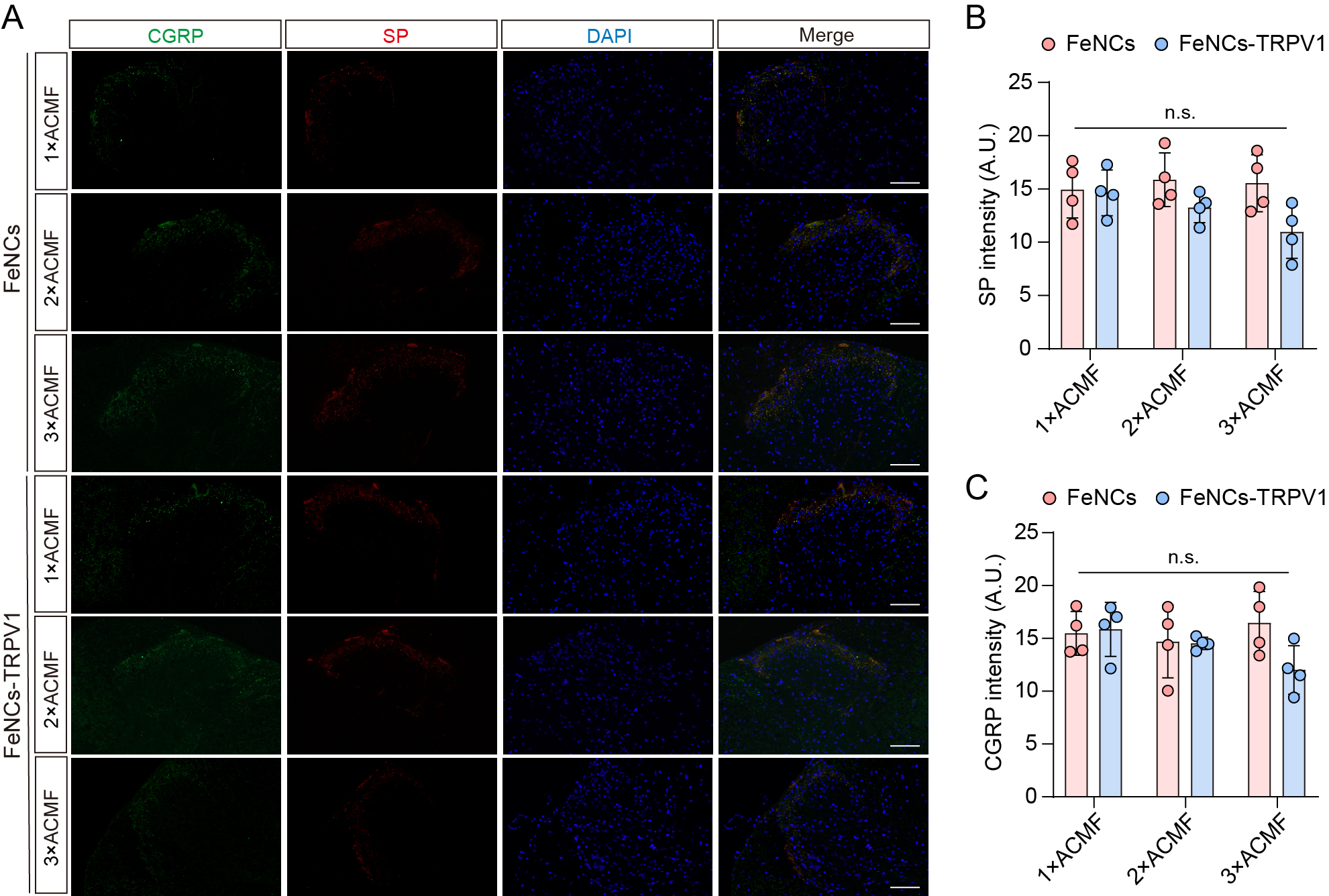


**Figure S12. Effects of ACMF exprosure alone on neuropeptide release in spinal cord.** (A) Representative images showing CGRP and SP immunostaining in spinal cord. Scale bar = 50 µm. (B, C) Quantification of SP (B) and CGRP (C) fluorescence intensity (arbitrary units, A.U.). Data are presented as the mean ± SD. Statistical analysis were performed using two-way ANOVA followed by Tukey’s test (*n* = 4 per group).


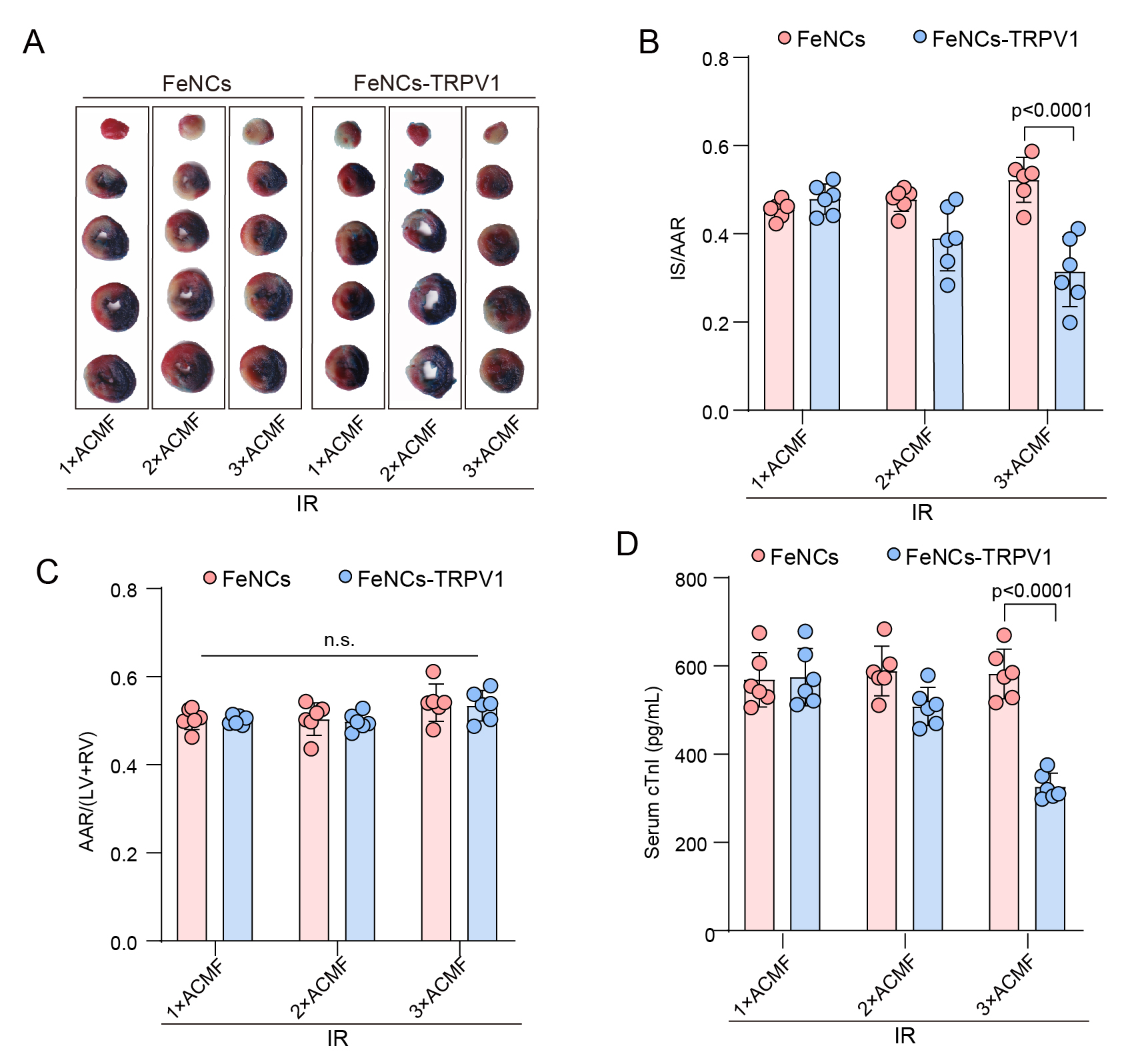


**Figure S13. Effects of different cycles of ACMF exposure on myocardial ischemia-reperfusion injury.** (A) Representative images of heart sections stained by TTC and Evans blue. (B) Infarct size (IS) is expressed as the ratio to area at risk (AAR). (C) The ratio of AAR to left ventricle (LV) and right ventricle (RV). (D) Serum cardiac troponin I (cTnI) levels measured by ELISA. Data represent the mean ± SD. Statistical analysis was performed using two-way ANOVA followed by Tukey’s test (*n* = 6 per group).

**Supplemental tables**

Supplemental Table S1. Arrhythmic events ( ± s)

| Groups | | Arrhythmia events and HR | Baseline | Ischemia | Reperfusion |
| --- | --- | --- | --- | --- | --- |
| Sham | FeNCs | PVCs (number of times) | 0 | 37 ± 17.1 | 21.3 ± 10.7 |
|  |  | VT (sec) | 0 | 0 | 0.17 ± 0.4 |
|  |  | VF (sec) | 0 | 0 | 0 |
|  |  | HR (beats/min) | 368 ± 19 | 358 ± 15 | 355 ± 17 |
|  | FeNCs-TRPV1 | PVCs (number of times) | 0 | 34.8 ± 12.3 | 27.5 ± 11.0 |
|  |  | VT (sec) | 0 | 0 | 0 |
|  |  | VF (sec) | 0 | 0 | 0 |
|  |  | HR (beats/min) | 370 ± 18 | 358 ± 17 | 362 ± 12 |
| IR | FeNCs | PVCs (number of times) | 0 | 33.8 ± 15.4 | 43.7 ± 23.5 |
|  |  | VT (sec) | 0 | 53.3 ± 15.3 | 76.3 ± 25.8 |
|  |  | VF (sec) | 0 | 0.4 ± 0.5 | 1.3 ± 1.0 |
|  |  | HR (beats/min) | 368 ± 18 | 320 ± 15 | 350 ± 13 |
|  | FeNCs-TRPV1 | PVCs (number of times) | 0 | 28.8 ± 14.7 | 57.5 ± 16.8 |
|  |  | VT (sec) | 0 | 14.8±8.5 | 29.3 ± 21.4 |
|  |  | VF (sec) | 0 | 0 | 0.17 ± 0.4 |
|  |  | Heart rates | 381 ± 18 | 326 ± 16 | 364 ± 12 |

PVC, premature ventricular contraction; VT, ventricular tachycardia; VF, ventricular fibrillation; Data are presented as mean ± SD, *n* = 6 per group.

Supplemental Table S2. The detail information for the number of animals in each group

| Experimental group | Randomized/tested | Excluded | | |
| --- | --- | --- | --- | --- |
|  |  | Unscheduled  Death | Intrathoracic  infection | Motor or sensory deficit |
| Sham+FeNCs | 19/17 | 0 | 2 | 0 |
| Sham+FeNCs-TRPV1 | 19/17 | 1 | 0 | 1 |
| IR+FeNCs | 20/17 | 1 | 1 | 1 |
| IR+FeNCs-TRPV1 | 19/17 | 2 | 0 | 0 |
| Sham | 17/17 | 0 | 0 | 0 |
| IR | 20/17 | 3 | 0 | 0 |
| IPC | 18/17 | 1 | 0 | 0 |
| Sham+ACMF | 17/17 | 0 | 0 | 0 |
| IR+ACMF | 19/17 | 2 | 0 | 0 |
| Control+FeNCs | 9/9 | 0 | 0 | 0 |
| Control+FeNCs-TRPV1 | 10/9 | 0 | 1 | 0 |
| 1×ACMF+FeNCs | 11/9 | 0 | 0 | 2 |
| 1×ACMF+FeNCs-TRPV1 | 11/9 | 1 | 1 | 0 |
| 2×ACMF+FeNCs | 10/9 | 0 | 1 | 0 |
| 2×ACMF+FeNCs-TRPV1 | 11/9 | 1 | 0 | 1 |
| 3×ACMF+FeNCs | 11/9 | 1 | 0 | 1 |
| 3×ACMF+FeNCs-TRPV1 | 10/9 | 0 | 1 | 0 |
| 1×ACMF+IR+FeNCs | 12/9 | 2 | 1 | 0 |
| 1×ACMF+IR+FeNCs-TRPV1 | 11/9 | 1 | 0 | 1 |
| 2×ACMF+IR+FeNCs | 12/9 | 1 | 1 | 1 |
| 2×ACMF+IR+FeNCs-TRPV1 | 11/9 | 2 | 0 | 0 |
| Total randomized/tested | 297/261 | 19 | 9 | 8 |
